# Supplementary material for: From Healer to Harmer: Preparing Senior Medical Students for Patient Harm Events in a Transition-to-Residency Course
Source: MedEdPORTAL. 2024 Dec 26;20:11473. doi: 10.15766/mep_2374-8265.11473 (PMC11669734; doi:10.15766/mep_2374-8265.11473)
Supplement: Supplementary file 1 — Pre- and Postsurvey.docxSecond Casualty Phenomenon.pptxInstructions for Residents.docxStudent Small-Group Prompts.docxCoping with Complications.pptxStudent Role-Play Instructions.docxWorkshop Facilitator Guide and Schedule.docx [file mep_2374-8265.11473-s001.zip › E. Coping with Complications.pptx]

## Slide 1
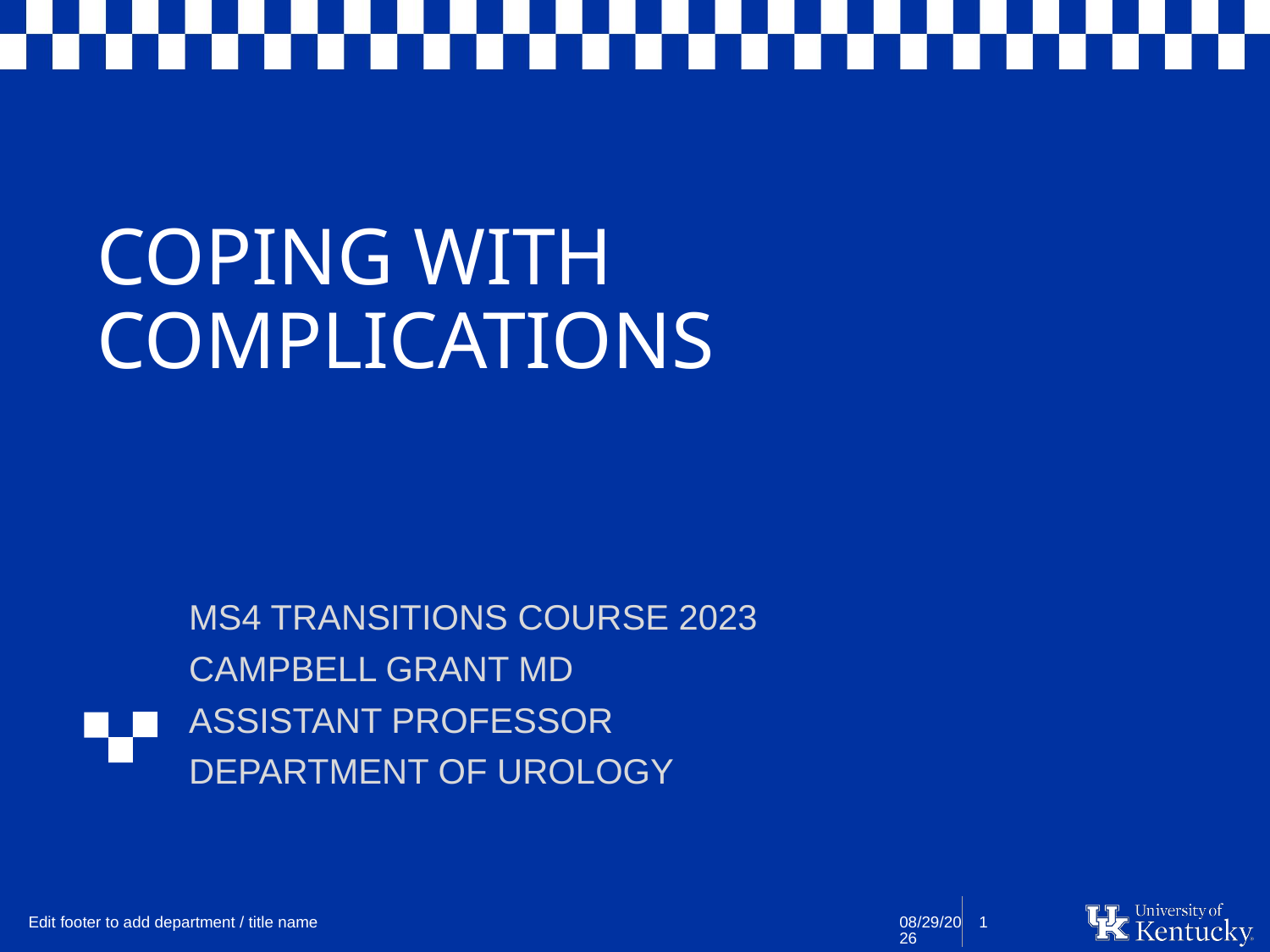

# Coping with Complications
MS4 Transitions course 2023
Campbell Grant MD
Assistant Professor
Department of Urology
7/12/2024
1
Edit footer to add department / title name

## Slide 2
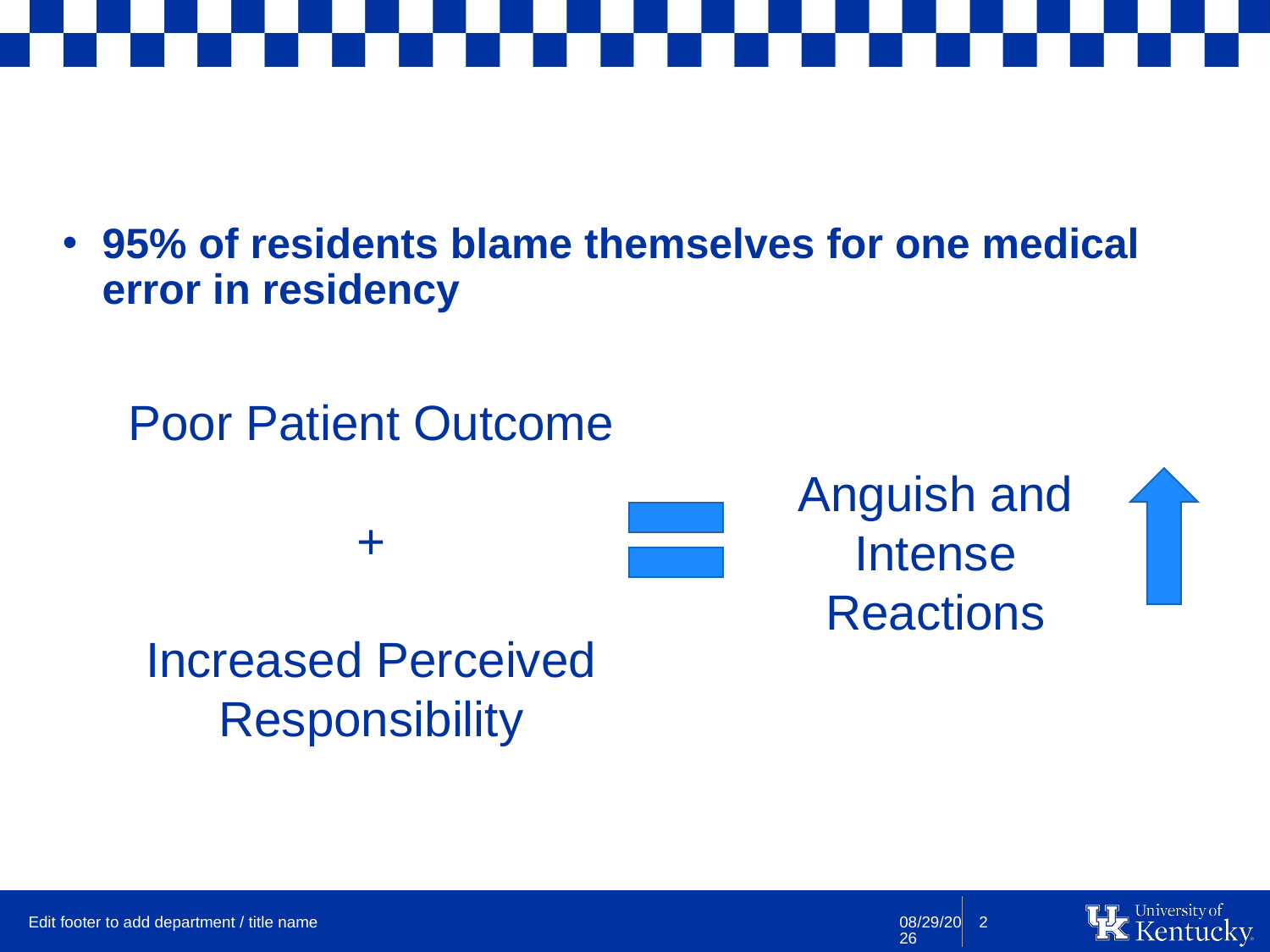

95% of residents blame themselves for one medical error in residency
Poor Patient Outcome
+
Increased Perceived Responsibility
Anguish and Intense Reactions
7/12/2024
2
Edit footer to add department / title name

## Slide 3
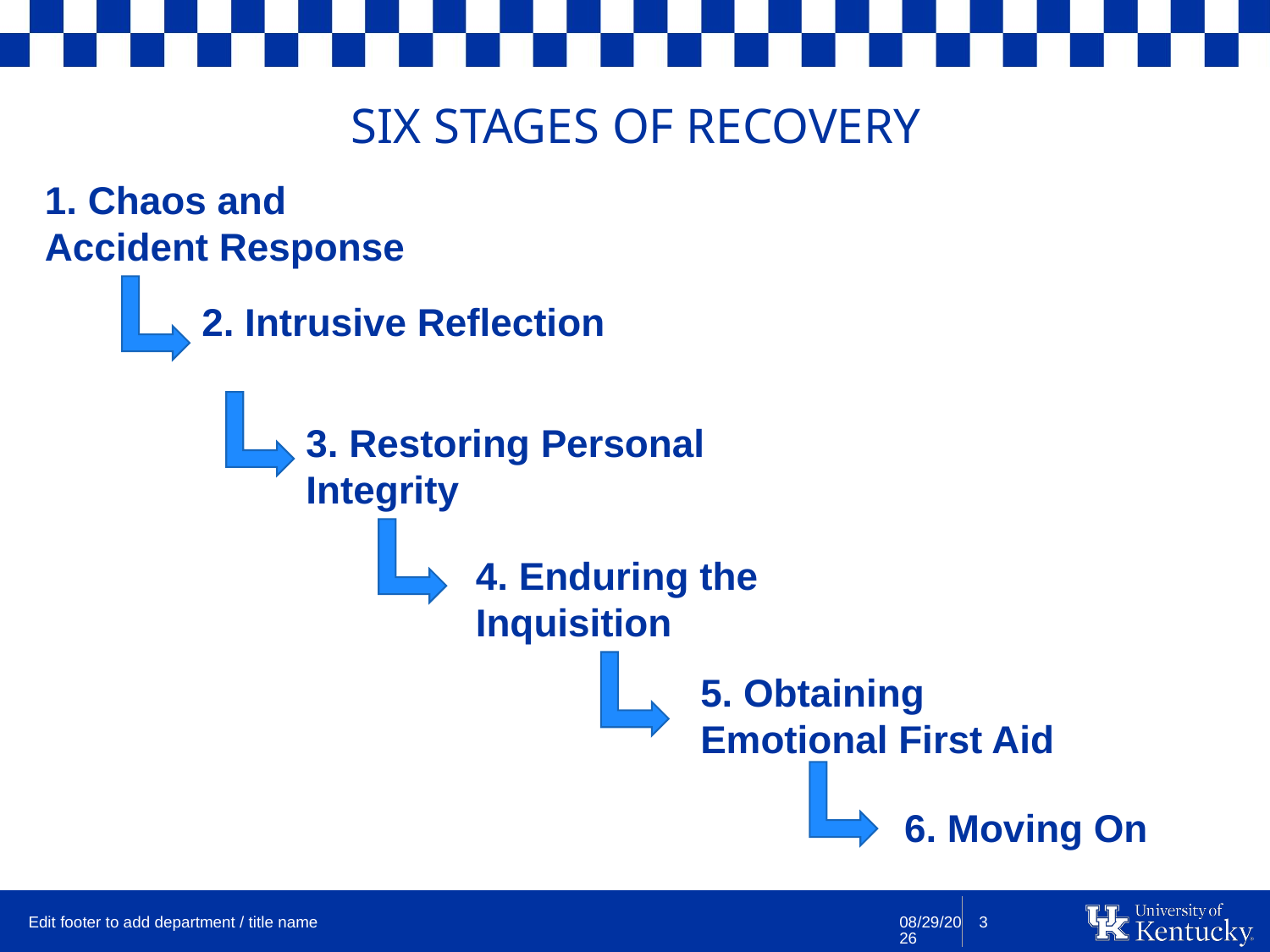

# Six Stages of Recovery
1. Chaos and Accident Response
2. Intrusive Reflection
3. Restoring Personal Integrity
4. Enduring the Inquisition
5. Obtaining Emotional First Aid
6. Moving On
7/12/2024
3
Edit footer to add department / title name

## Slide 4
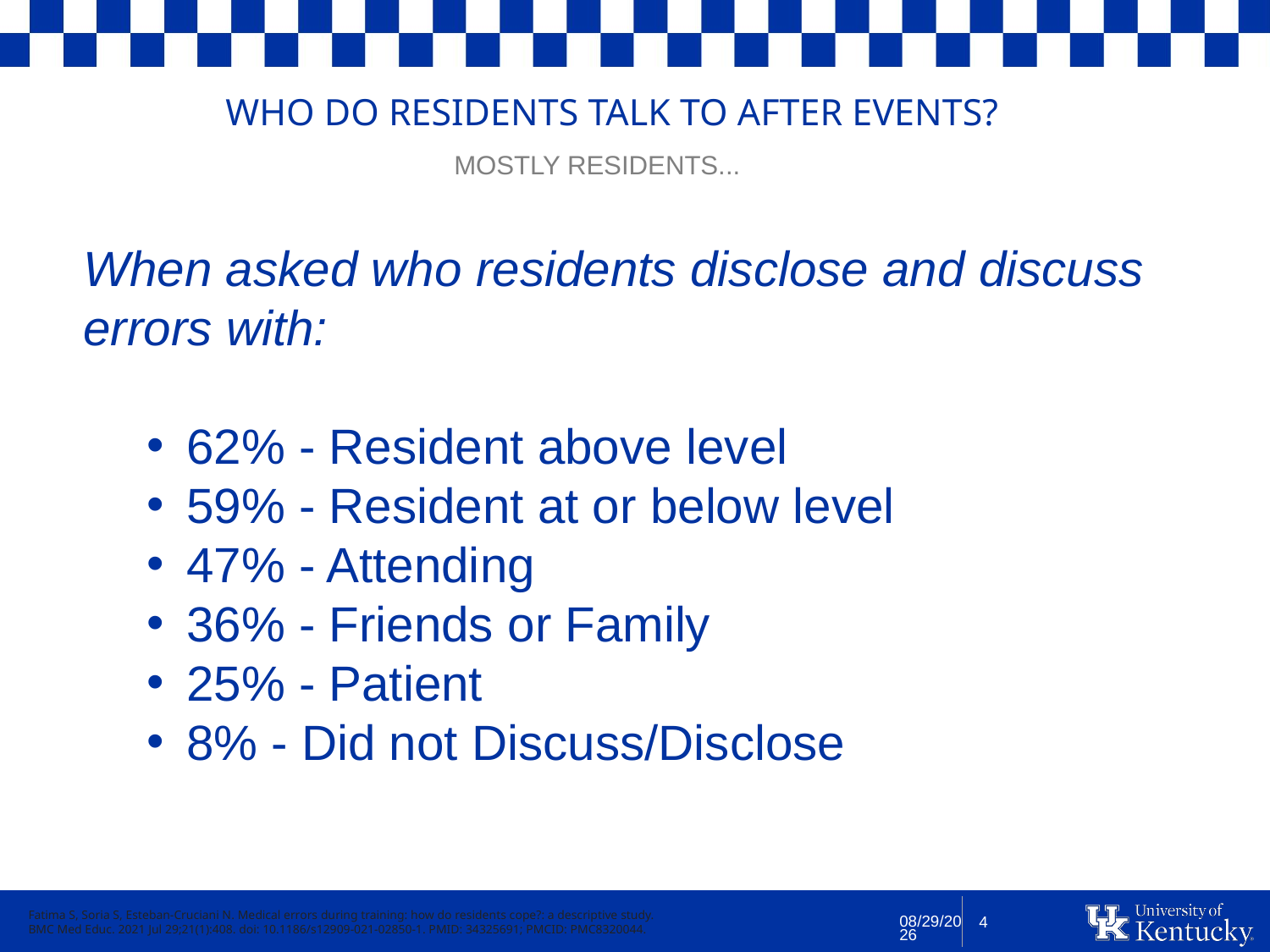

# Who do residents talk to after events?
Mostly residents...
When asked who residents disclose and discuss errors with:
62% - Resident above level
59% - Resident at or below level
47% - Attending
36% - Friends or Family
25% - Patient
8% - Did not Discuss/Disclose
7/12/2024
4
Fatima S, Soria S, Esteban-Cruciani N. Medical errors during training: how do residents cope?: a descriptive study. BMC Med Educ. 2021 Jul 29;21(1):408. doi: 10.1186/s12909-021-02850-1. PMID: 34325691; PMCID: PMC8320044.

## Slide 5
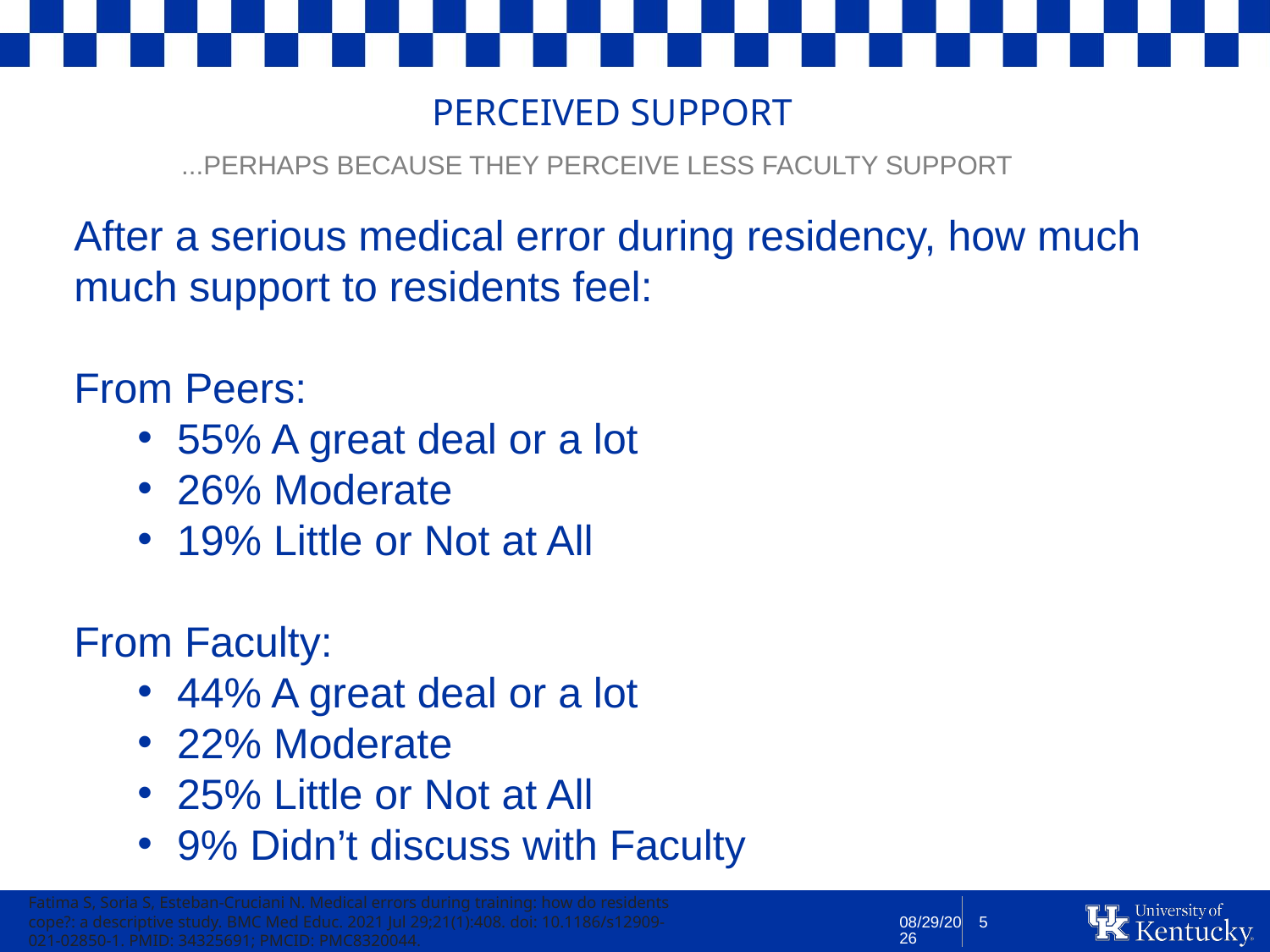

# Perceived support
...perhaps because they perceive less faculty support
After a serious medical error during residency, how much much support to residents feel:
From Peers:
55% A great deal or a lot
26% Moderate
19% Little or Not at All
From Faculty:
44% A great deal or a lot
22% Moderate
25% Little or Not at All
9% Didn’t discuss with Faculty
7/12/2024
5
Fatima S, Soria S, Esteban-Cruciani N. Medical errors during training: how do residents cope?: a descriptive study. BMC Med Educ. 2021 Jul 29;21(1):408. doi: 10.1186/s12909-021-02850-1. PMID: 34325691; PMCID: PMC8320044.

## Slide 6
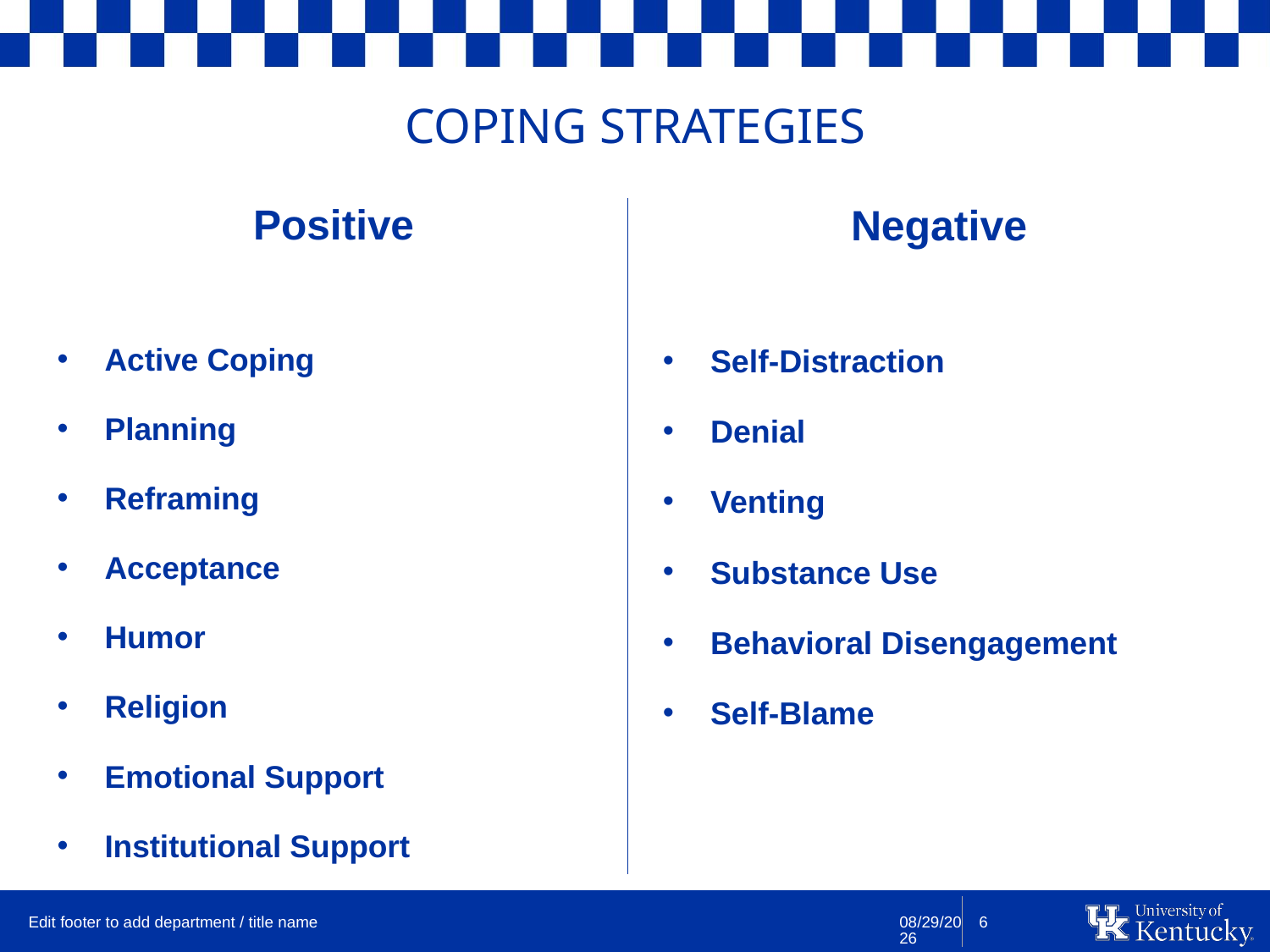

# Coping Strategies
Negative
Self-Distraction
Denial
Venting
Substance Use
Behavioral Disengagement
Self-Blame
Positive
Active Coping
Planning
Reframing
Acceptance
Humor
Religion
Emotional Support
Institutional Support
7/12/2024
6
Edit footer to add department / title name

## Slide 7
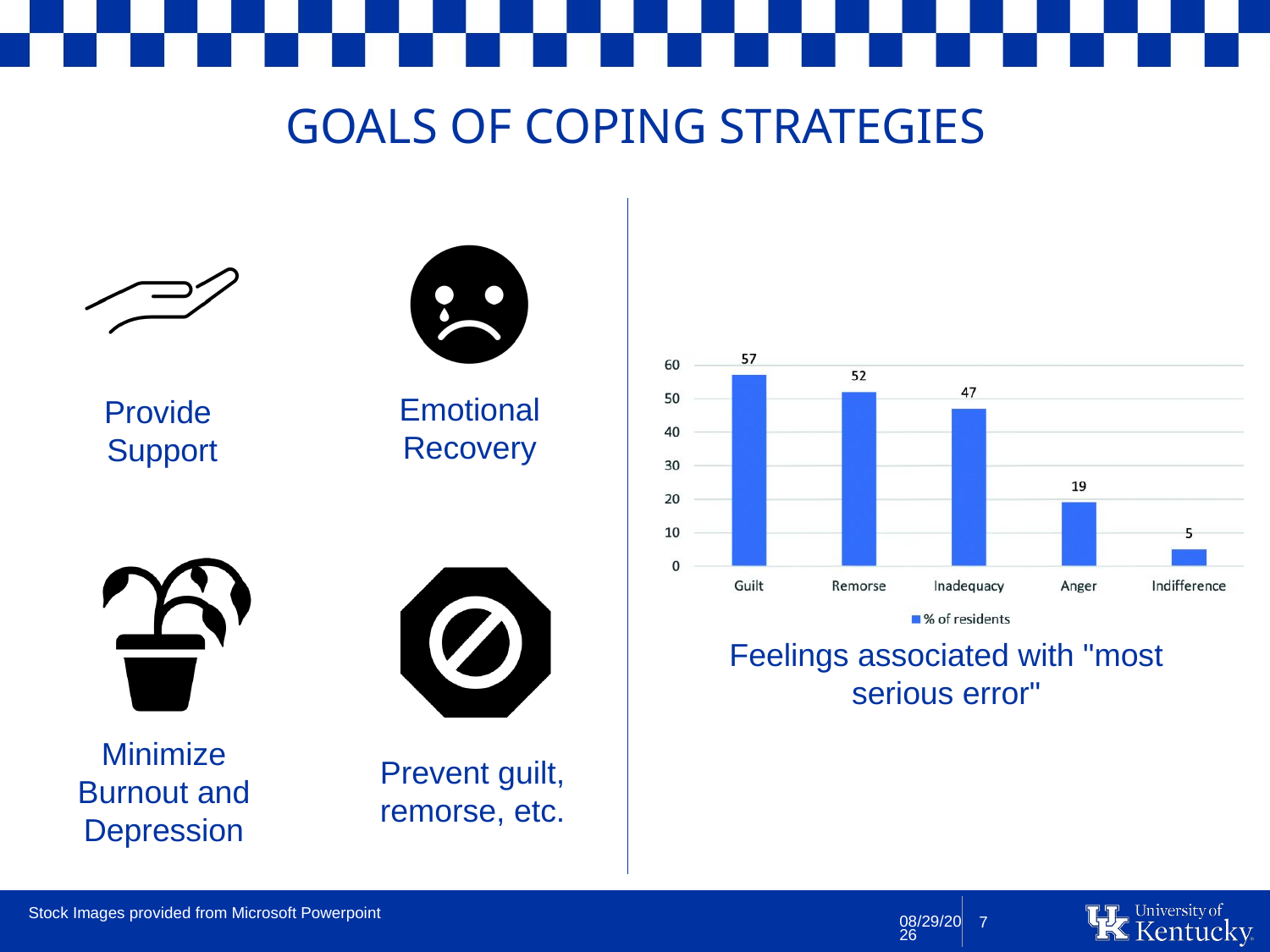

# Goals of Coping Strategies
Emotional Recovery
Provide
Support
Feelings associated with "most serious error"
Minimize Burnout and Depression
Prevent guilt, remorse, etc.
7/12/2024
7
Stock Images provided from Microsoft Powerpoint

## Slide 8
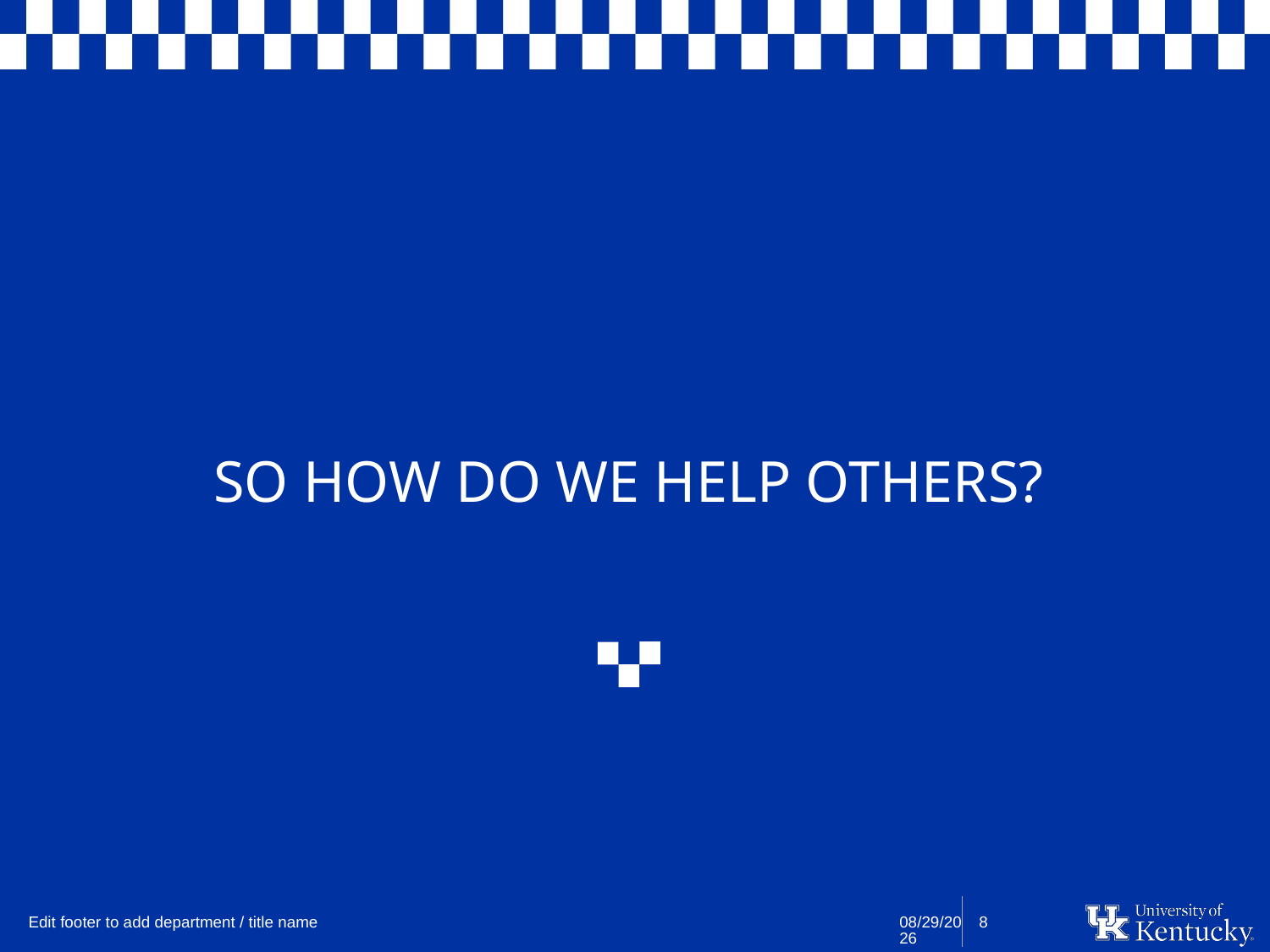

# So how do we help others?
7/12/2024
8
Edit footer to add department / title name

## Slide 9
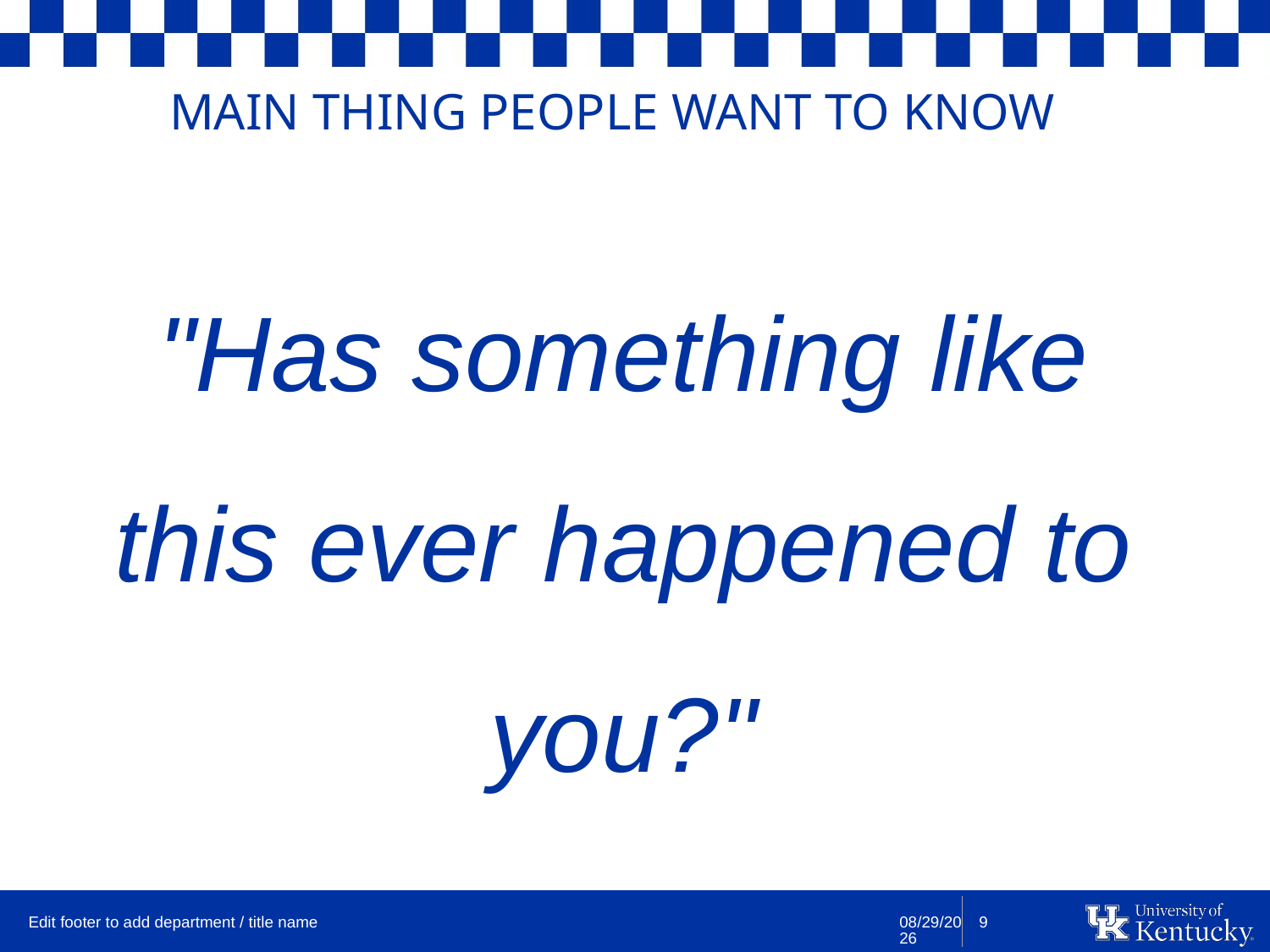

# Main thing people want to know
"Has something like this ever happened to you?"
7/12/2024
9
Edit footer to add department / title name

## Slide 10
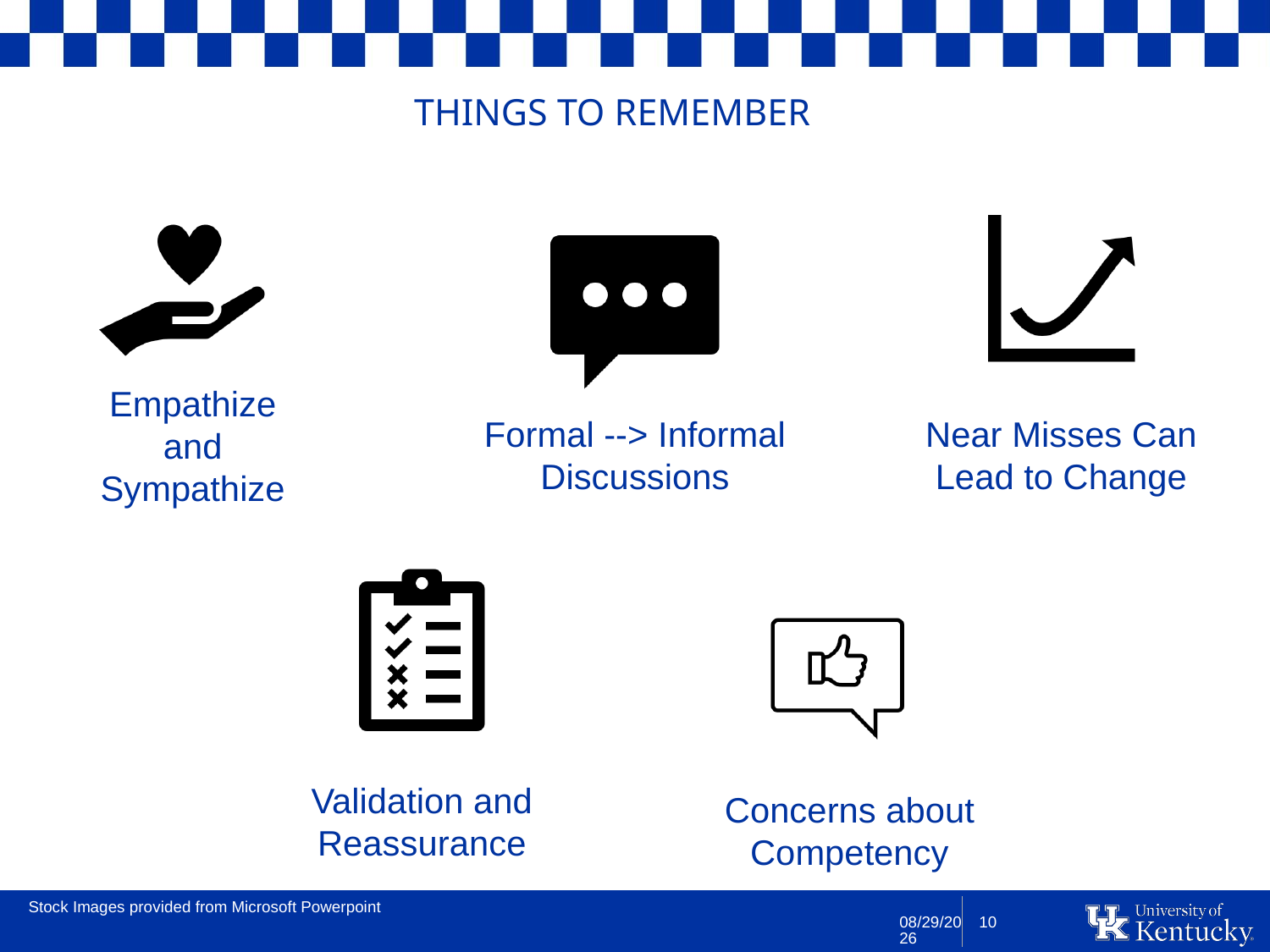

# Things to remember
Empathize and Sympathize
Formal --> Informal Discussions
Near Misses Can Lead to Change
Validation and Reassurance
Concerns about Competency
7/12/2024
10
Stock Images provided from Microsoft Powerpoint

## Slide 11
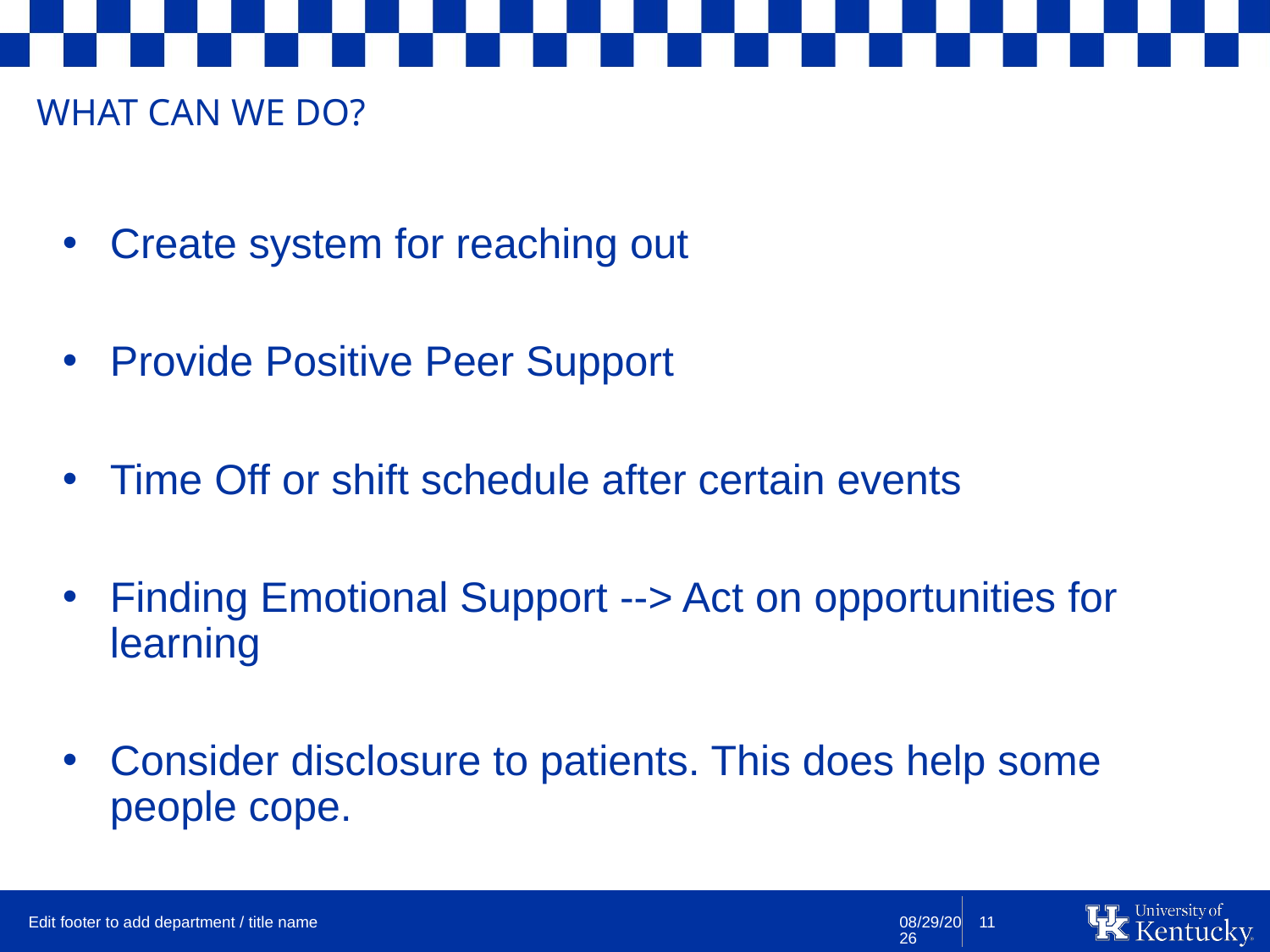

# What Can we do?
Create system for reaching out
Provide Positive Peer Support
Time Off or shift schedule after certain events
Finding Emotional Support --> Act on opportunities for learning
Consider disclosure to patients. This does help some people cope.
7/12/2024
11
Edit footer to add department / title name

## Slide 12
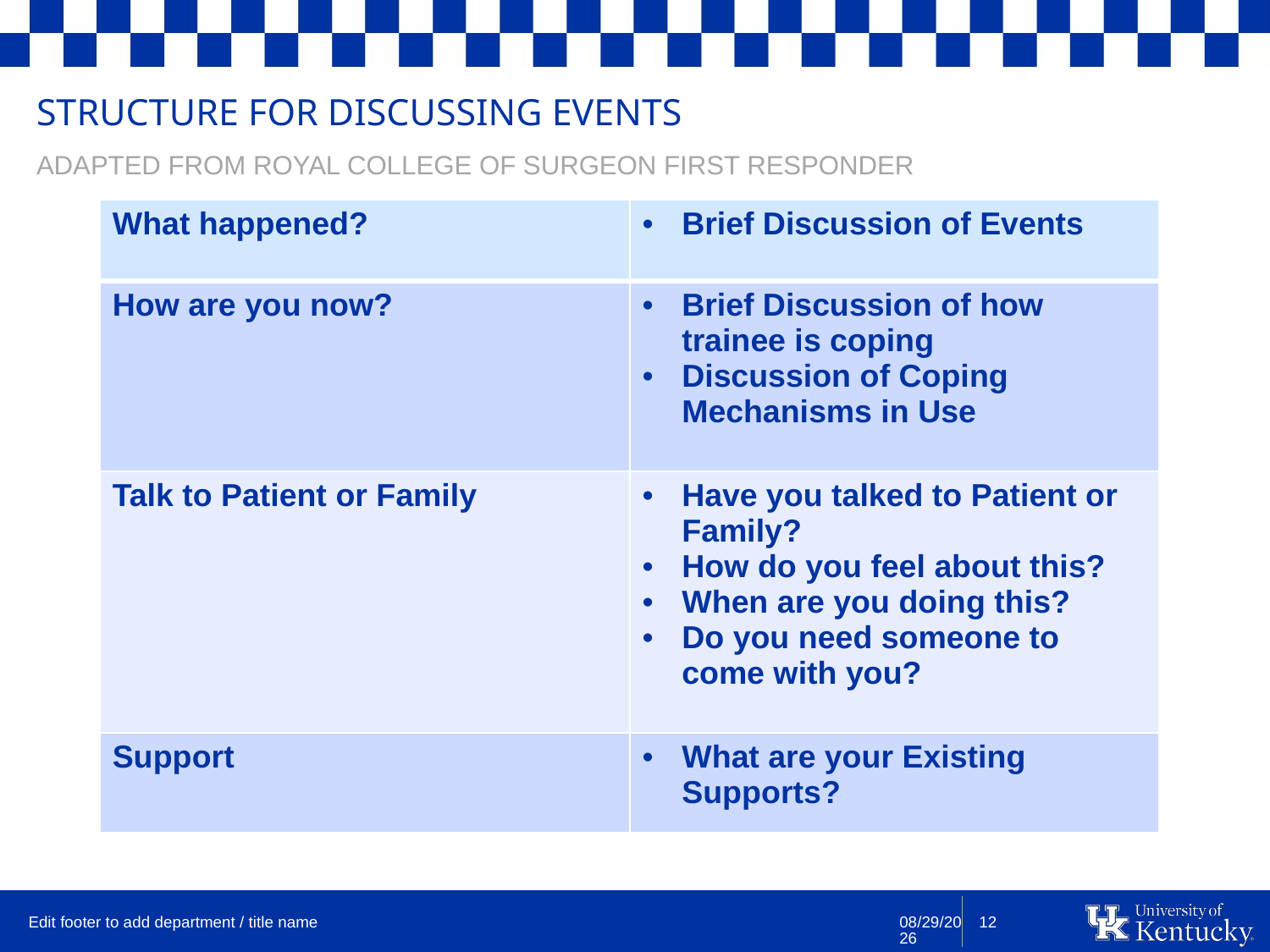

# Structure for discussing events
Adapted from Royal College of Surgeon First responder
| What happened? | Brief Discussion of Events |
| --- | --- |
| How are you now? | Brief Discussion of how trainee is coping Discussion of Coping Mechanisms in Use |
| Talk to Patient or Family | Have you talked to Patient or Family? How do you feel about this? When are you doing this? Do you need someone to come with you? |
| Support | What are your Existing Supports? |
7/12/2024
12
Edit footer to add department / title name

## Slide 13
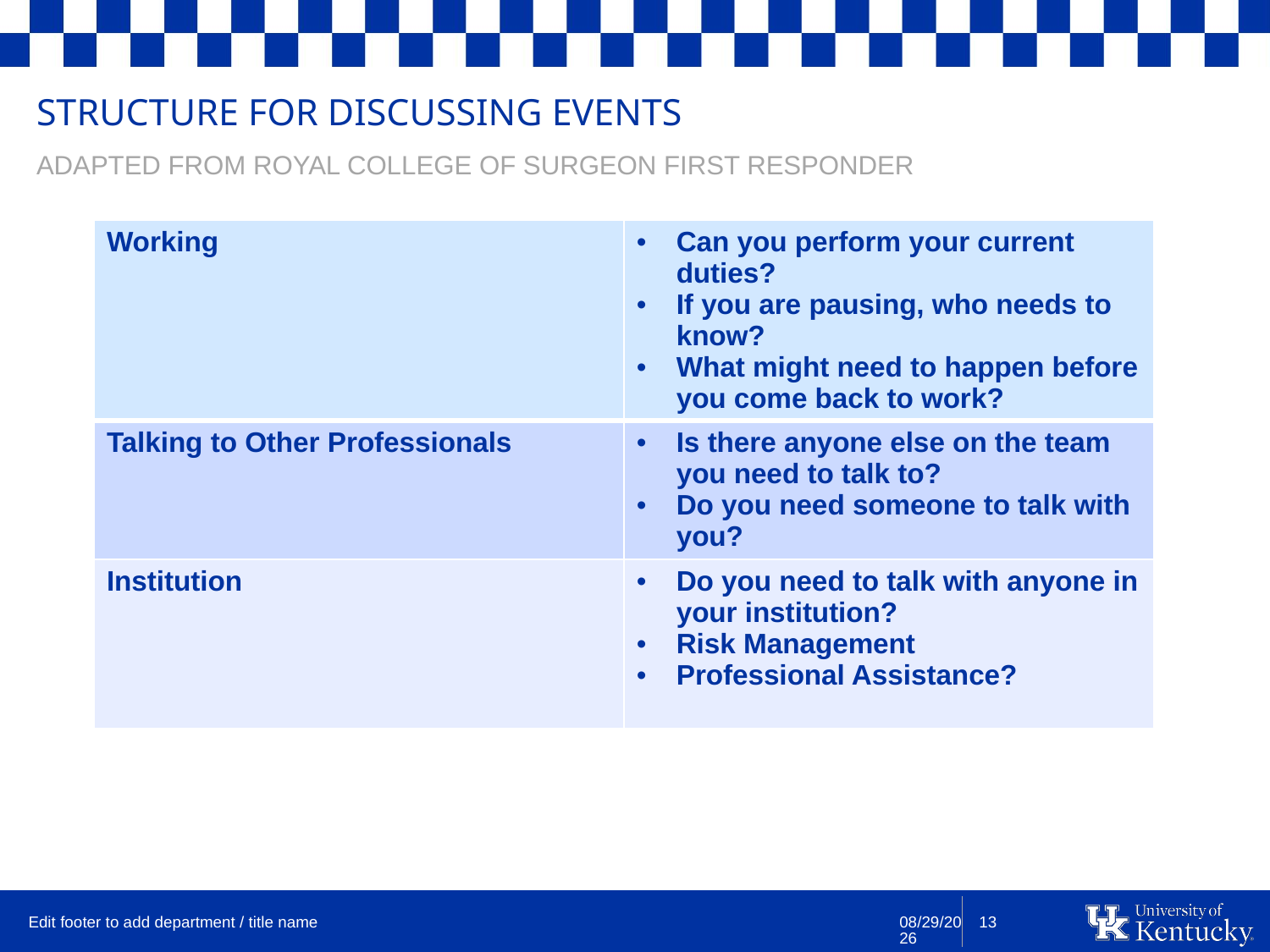

# Structure for discussing events
Adapted from Royal College of Surgeon First responder
| Working | Can you perform your current duties? If you are pausing, who needs to know? What might need to happen before you come back to work? |
| --- | --- |
| Talking to Other Professionals | Is there anyone else on the team you need to talk to? Do you need someone to talk with you? |
| Institution | Do you need to talk with anyone in your institution? Risk Management Professional Assistance? |
7/12/2024
13
Edit footer to add department / title name
