# Supplementary material for: From Healer to Harmer: Preparing Senior Medical Students for Patient Harm Events in a Transition-to-Residency Course
Source: MedEdPORTAL. 2024 Dec 26;20:11473. doi: 10.15766/mep_2374-8265.11473 (PMC11669734; doi:10.15766/mep_2374-8265.11473)
Supplement: Supplementary file 1 — Pre- and Postsurvey.docxSecond Casualty Phenomenon.pptxInstructions for Residents.docxStudent Small-Group Prompts.docxCoping with Complications.pptxStudent Role-Play Instructions.docxWorkshop Facilitator Guide and Schedule.docx [file mep_2374-8265.11473-s001.zip › C. Instructions for Residents.docx]

**The resident panel should consist of 2-3 residents. Residents can be recruited by asking if any residents have been involved in patient harm events that negatively affected them that they are willing to discuss in an open forum. Program directors are often helpful in identifying residents who may be good panelists.**

**Instructions for Residents (to be given to the residents prior to the panel)**

Think of when you felt responsible or were involved in a patient harm event. Choose an event that was either highly impactful or one from which you learned a great deal.

a) In 5 minutes, share the story of this event

b) Next, share how you felt when you discovered the error

c) How did the error affect you in-side and out-side of work

d) How did you process or not process the emotions that coincided with the event?

e) What would you do or do you do differently now in terms of emotional processing?
