# Supplementary material for: From Healer to Harmer: Preparing Senior Medical Students for Patient Harm Events in a Transition-to-Residency Course
Source: MedEdPORTAL. 2024 Dec 26;20:11473. doi: 10.15766/mep_2374-8265.11473 (PMC11669734; doi:10.15766/mep_2374-8265.11473)
Supplement: Supplementary file 1 — Pre- and Postsurvey.docxSecond Casualty Phenomenon.pptxInstructions for Residents.docxStudent Small-Group Prompts.docxCoping with Complications.pptxStudent Role-Play Instructions.docxWorkshop Facilitator Guide and Schedule.docx [file mep_2374-8265.11473-s001.zip › A. Pre- and Postsurvey.docx]

| I am confident I can… | | | | | |
| --- | --- | --- | --- | --- | --- |
|  | Not Confident  At All | Somewhat Confident | Moderately Confident | Very Confident | Extremely Confident |
| Define Second Casualty Phenomenon | 🞏 | 🞏 | 🞏 | 🞏 | 🞏 |
| Utilize positive coping mechanisms to reduce anxiety after patient harm events | 🞏 | 🞏 | 🞏 | 🞏 | 🞏 |
| Utilize First Responder structure for discussing patient harm events | 🞏 | 🞏 | 🞏 | 🞏 | 🞏 |
| Help colleagues cope with patient harm events they caused | 🞏 | 🞏 | 🞏 | 🞏 | 🞏 |

***This survey should be given to students as they enter the classroom or during the introduction.***

| After this session, I am confident I can… | | | | | |
| --- | --- | --- | --- | --- | --- |
|  | Not Confident  At All | Somewhat Confident | Moderately Confident | Very Confident | Extremely Confident |
| Define Second Casualty Phenomenon | 🞏 | 🞏 | 🞏 | 🞏 | 🞏 |
| Utilize positive coping mechanisms to reduce anxiety after patient harm events | 🞏 | 🞏 | 🞏 | 🞏 | 🞏 |
| Utilize First Responder structure for discussing patient harm events | 🞏 | 🞏 | 🞏 | 🞏 | 🞏 |
| Help colleagues cope with patient harm events they caused | 🞏 | 🞏 | 🞏 | 🞏 | 🞏 |

***This survey can be given to students during the role play sessions and collected at the end of the session.***
