# Supplementary material for: From Healer to Harmer: Preparing Senior Medical Students for Patient Harm Events in a Transition-to-Residency Course
Source: MedEdPORTAL. 2024 Dec 26;20:11473. doi: 10.15766/mep_2374-8265.11473 (PMC11669734; doi:10.15766/mep_2374-8265.11473)
Supplement: Supplementary file 1 — Pre- and Postsurvey.docxSecond Casualty Phenomenon.pptxInstructions for Residents.docxStudent Small-Group Prompts.docxCoping with Complications.pptxStudent Role-Play Instructions.docxWorkshop Facilitator Guide and Schedule.docx [file mep_2374-8265.11473-s001.zip › B. Second Casualty Phenomenon.pptx]

## Slide 1
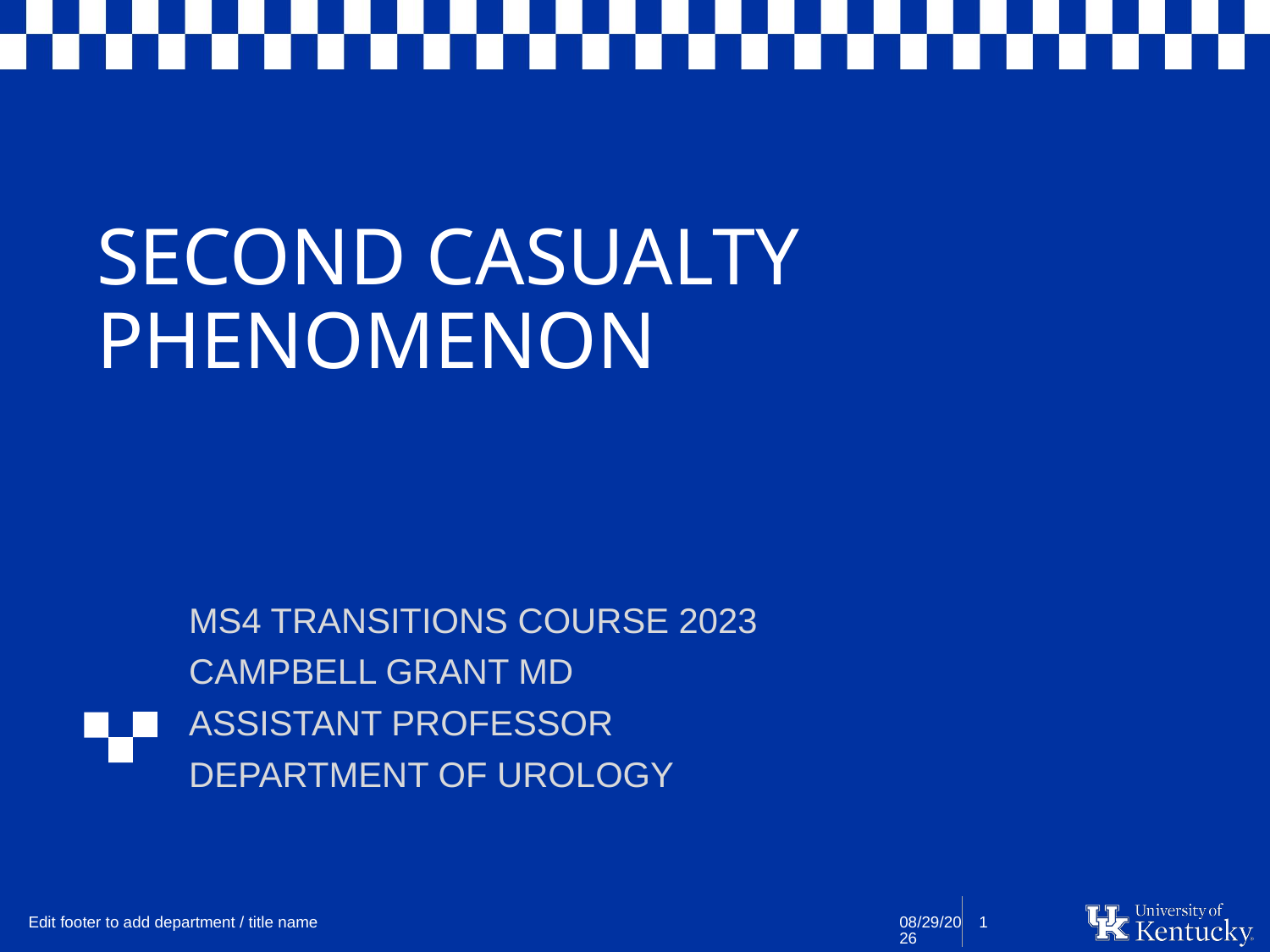

# Second Casualty phenomenon
MS4 TRANSITIONS COURSE 2023
CAMPBELL GRANT MD
ASSISTANT PROFESSOR
DEPARTMENT OF UROLOGY
7/12/2024
1
Edit footer to add department / title name

## Slide 2
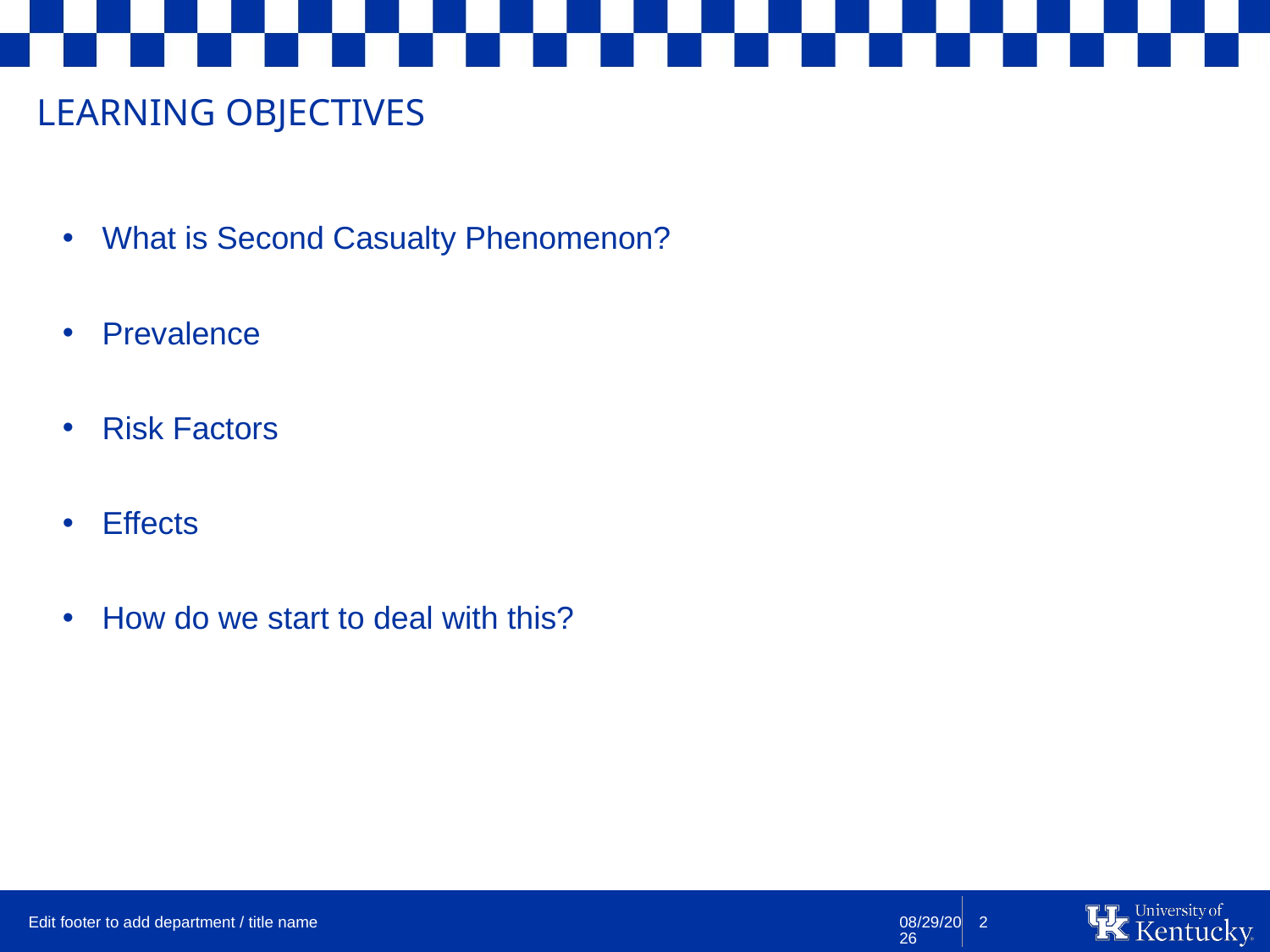

# Learning Objectives
What is Second Casualty Phenomenon?
Prevalence
Risk Factors
Effects
How do we start to deal with this?
7/12/2024
2
Edit footer to add department / title name

## Slide 3
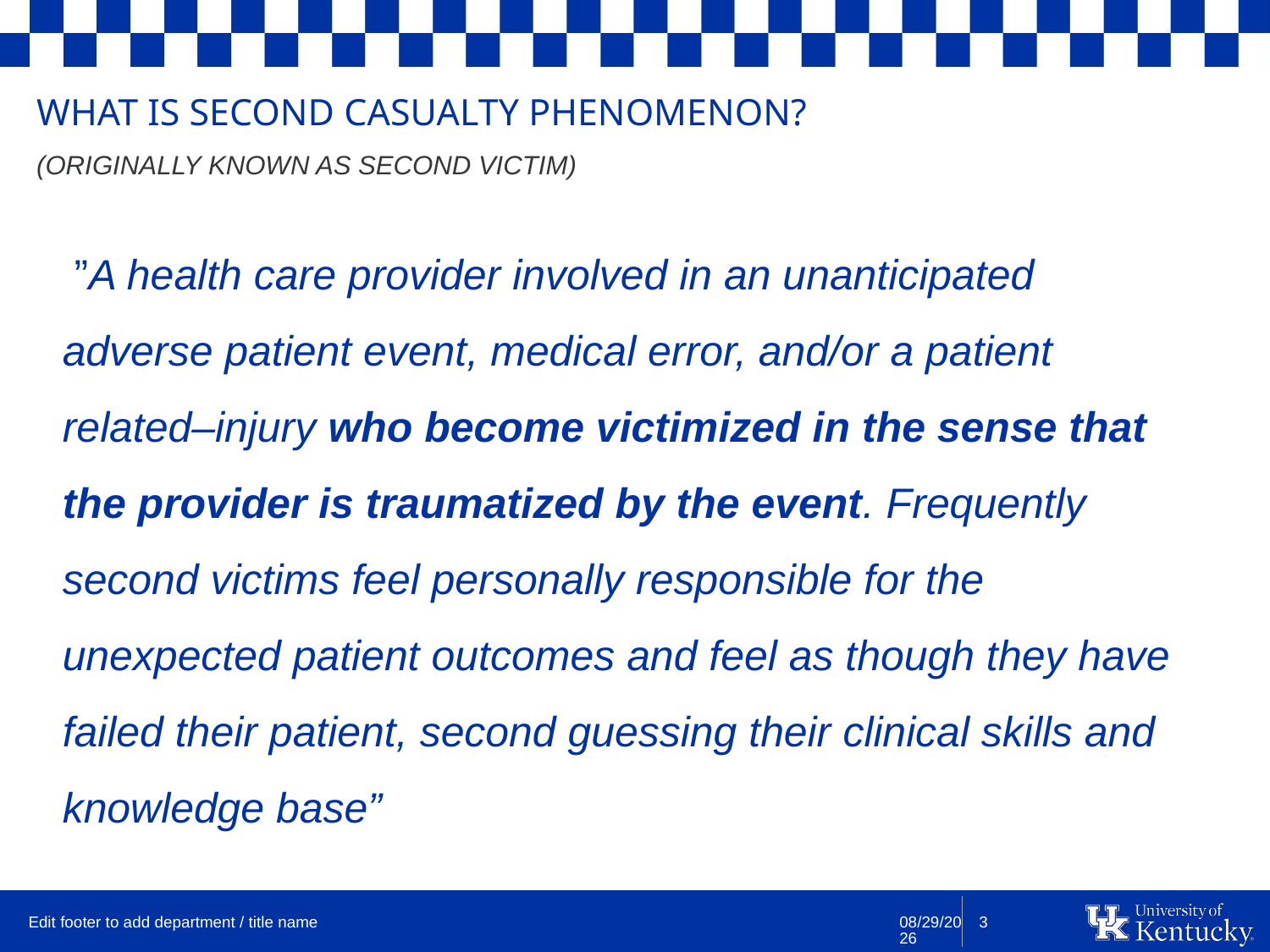

# What is second Casualty phenomenon?
(Originally known as second victim)
 ”A health care provider involved in an unanticipated adverse patient event, medical error, and/or a patient related–injury who become victimized in the sense that the provider is traumatized by the event. Frequently second victims feel personally responsible for the unexpected patient outcomes and feel as though they have failed their patient, second guessing their clinical skills and knowledge base”
7/12/2024
3
Edit footer to add department / title name

## Slide 4
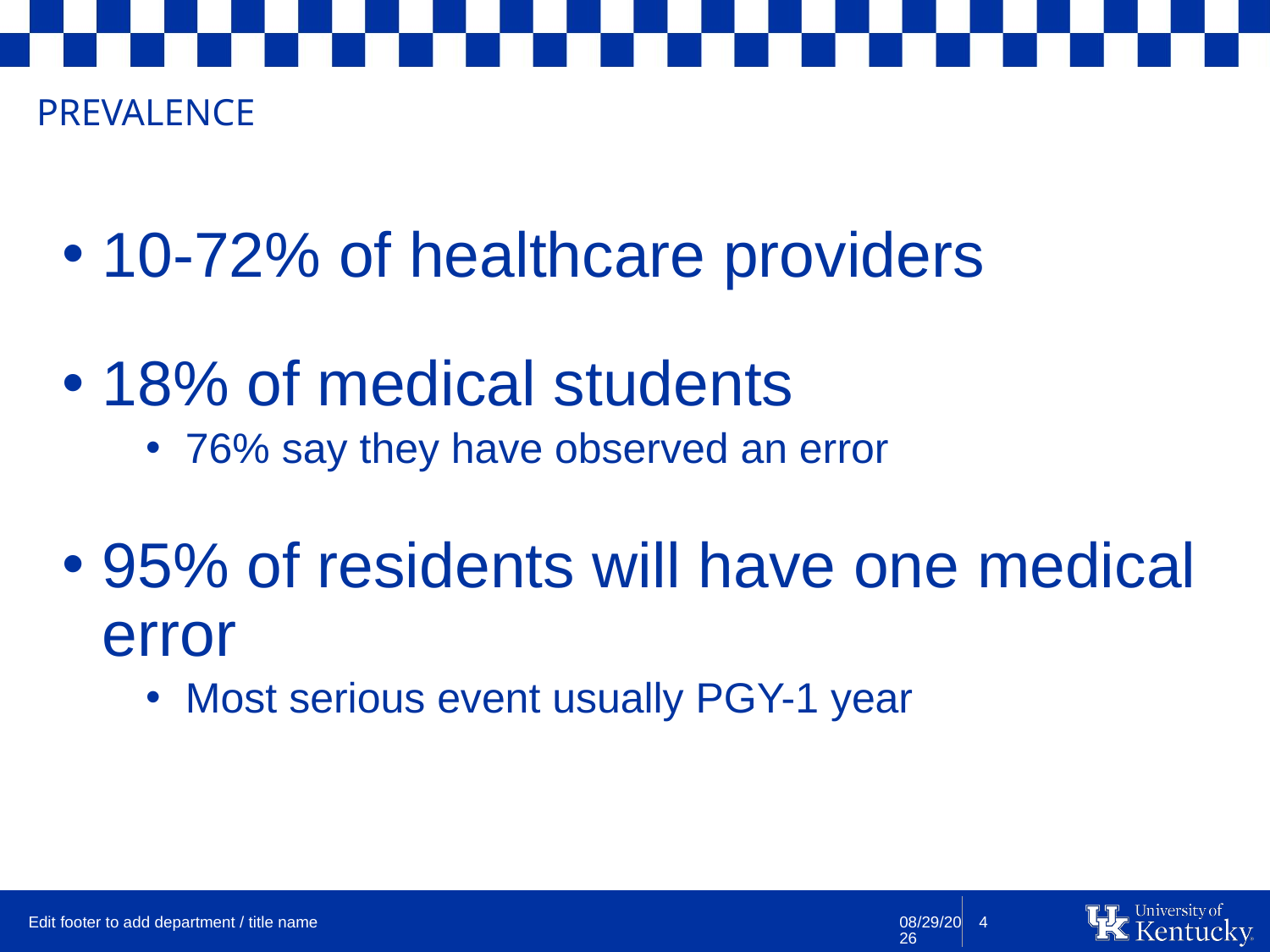

# Prevalence
10-72% of healthcare providers
18% of medical students
76% say they have observed an error
95% of residents will have one medical error
Most serious event usually PGY-1 year
7/12/2024
4
Edit footer to add department / title name

## Slide 5
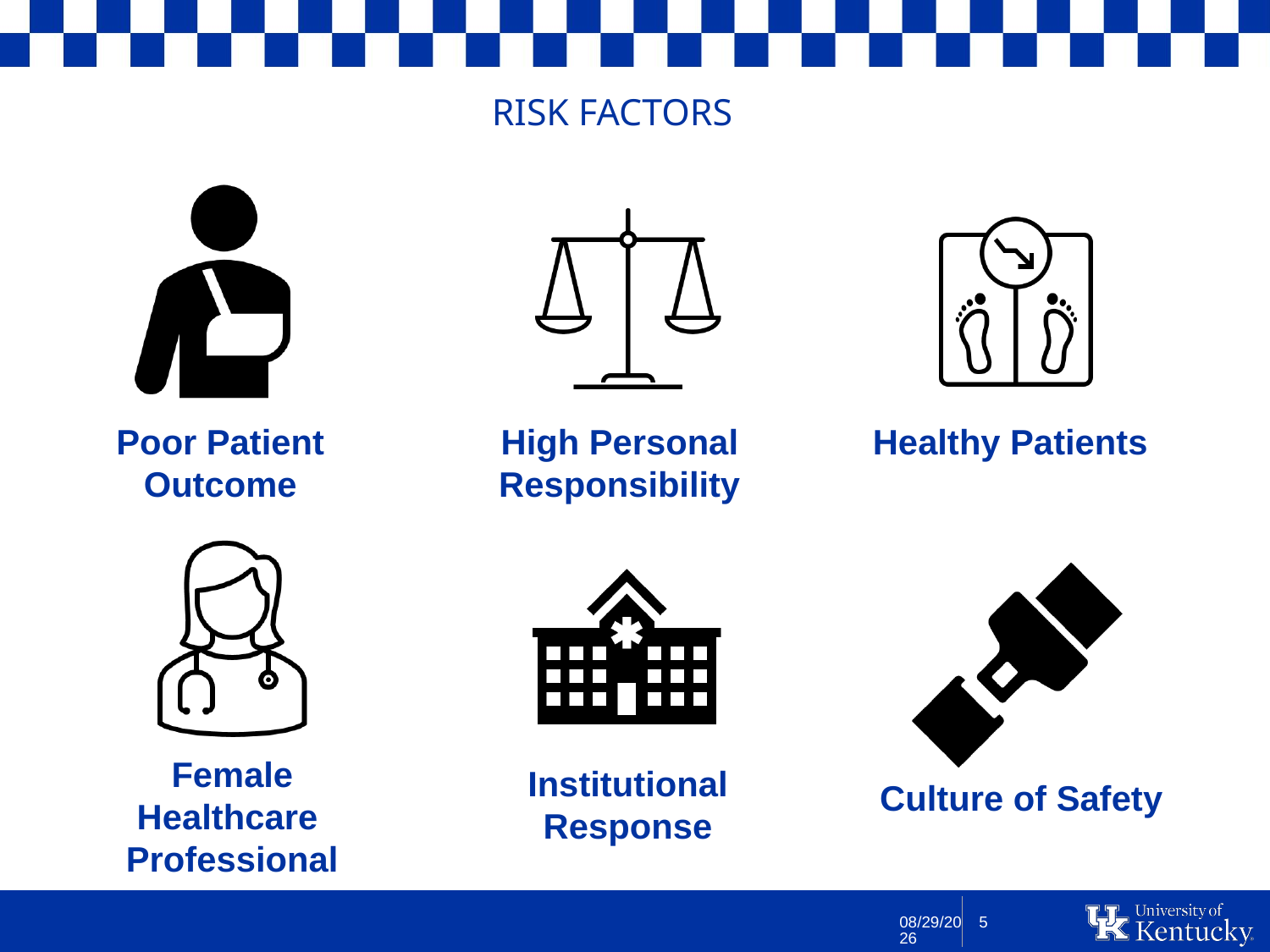

# Risk Factors
Healthy Patients
High Personal Responsibility
Poor Patient Outcome
Female Healthcare
Professional
Institutional Response
Culture of Safety
7/12/2024
5

## Slide 6
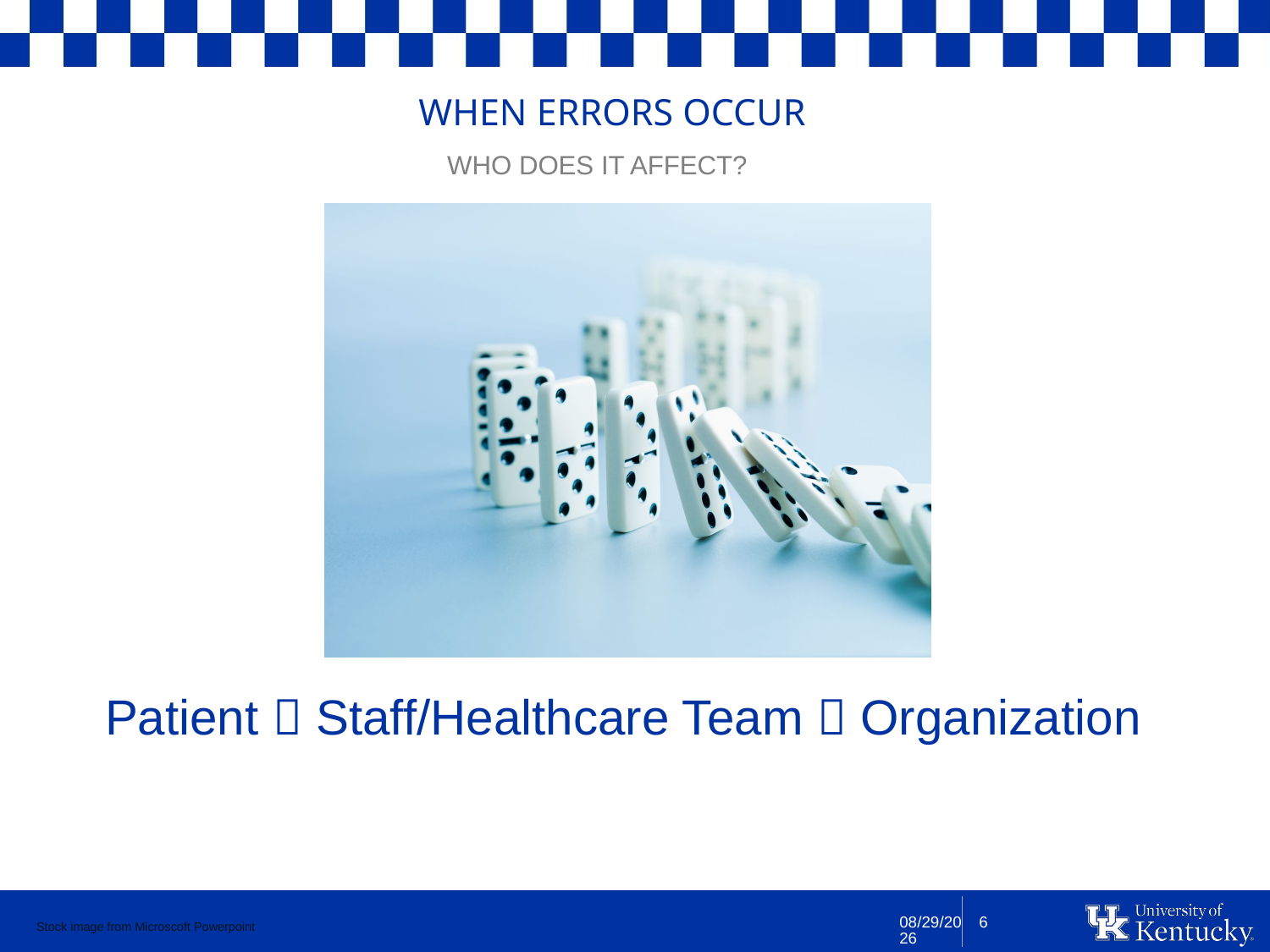

# When Errors Occur
Who does it Affect?
Patient  Staff/Healthcare Team  Organization
7/12/2024
6
Stock image from Microscoft Powerpoint

## Slide 7
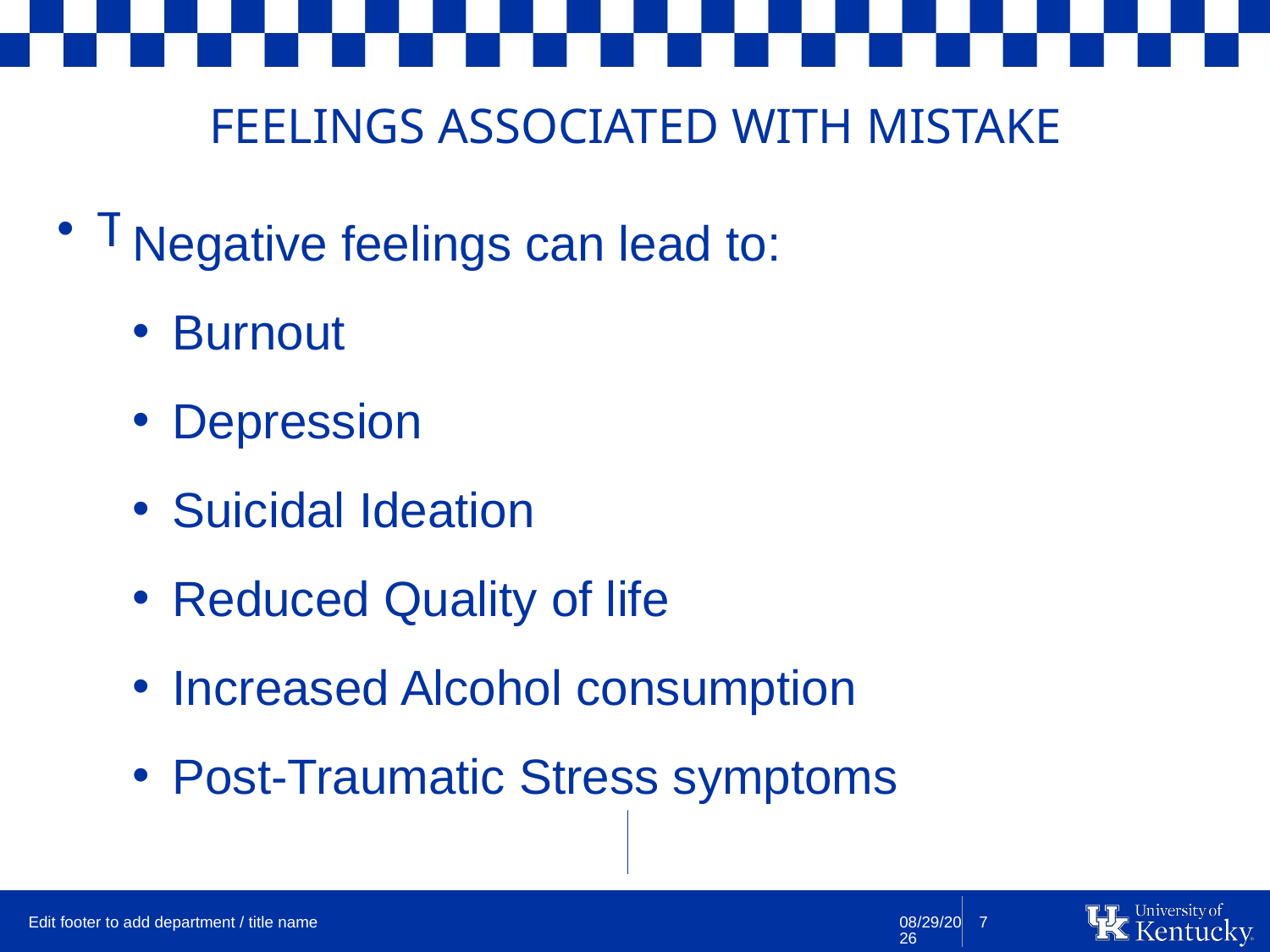

# Feelings Associated with Mistake
Negative feelings can lead to:
Burnout
Depression
Suicidal Ideation
Reduced Quality of life
Increased Alcohol consumption
Post-Traumatic Stress symptoms
The Good
Improve Relationships
Improved Care
The Bad
Guilt
Anxiety
Fatigue
Perfectionism Self-Doubt
“Should I tell anyone?”
7/12/2024
7
Edit footer to add department / title name

## Slide 8
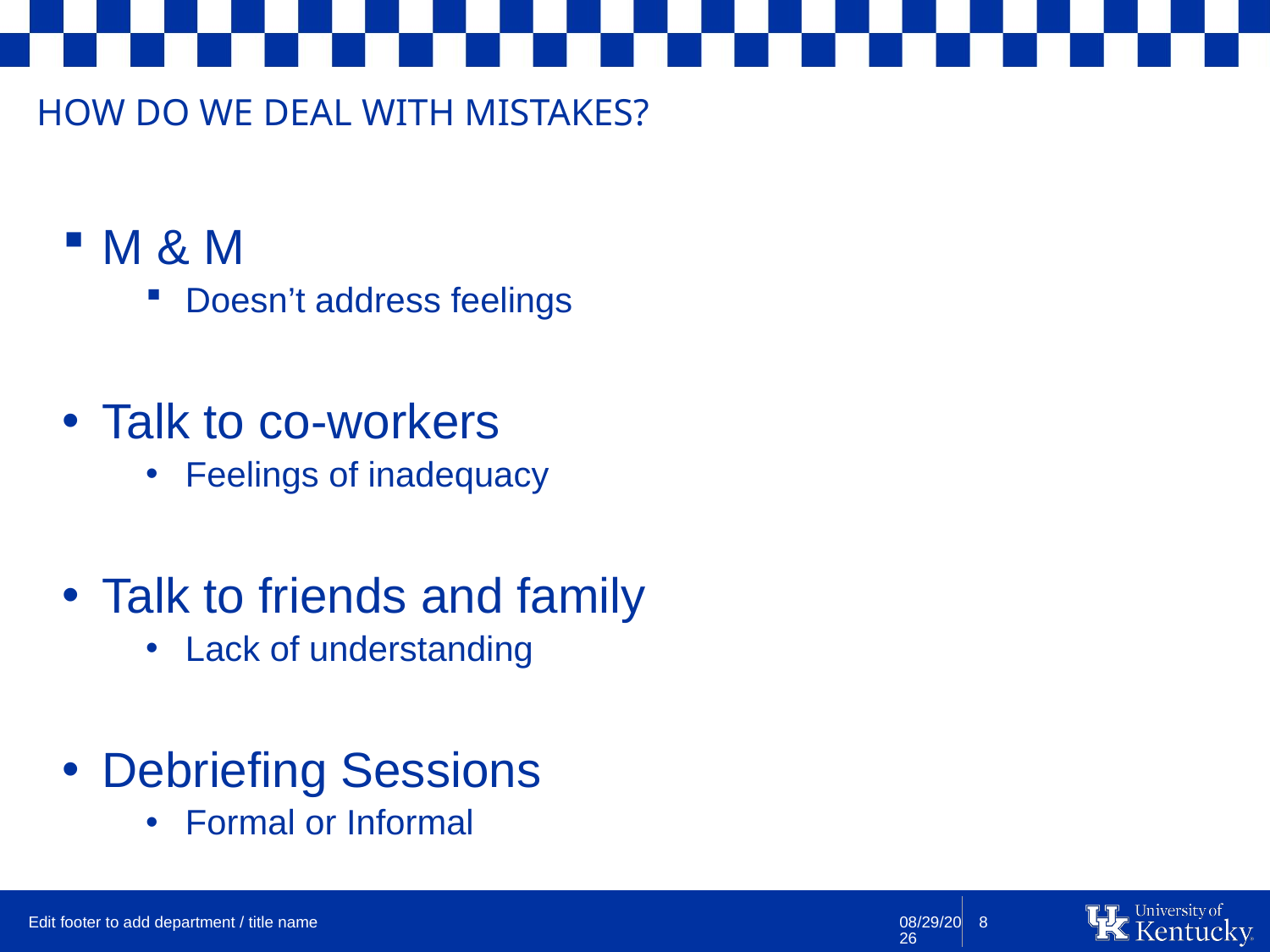

# How Do we deal with mistakes?
M & M
Doesn’t address feelings
Talk to co-workers
Feelings of inadequacy
Talk to friends and family
Lack of understanding
Debriefing Sessions
Formal or Informal
7/12/2024
8
Edit footer to add department / title name

## Slide 9
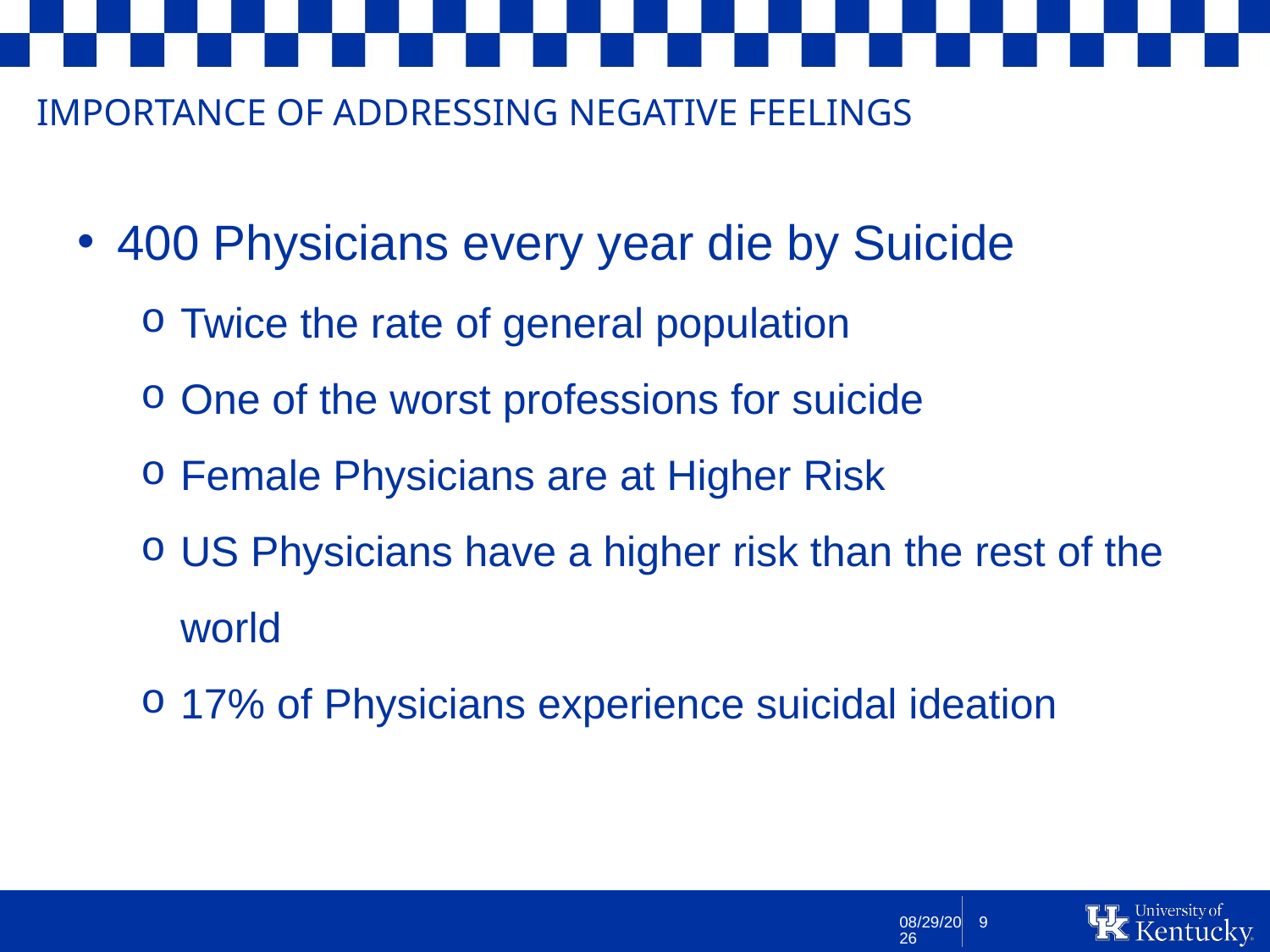

# Importance of addressing Negative Feelings
400 Physicians every year die by Suicide
Twice the rate of general population
One of the worst professions for suicide
Female Physicians are at Higher Risk
US Physicians have a higher risk than the rest of the world
17% of Physicians experience suicidal ideation
7/12/2024
9
