# Supplementary material for: From Healer to Harmer: Preparing Senior Medical Students for Patient Harm Events in a Transition-to-Residency Course
Source: MedEdPORTAL. 2024 Dec 26;20:11473. doi: 10.15766/mep_2374-8265.11473 (PMC11669734; doi:10.15766/mep_2374-8265.11473)
Supplement: Supplementary file 1 — Pre- and Postsurvey.docxSecond Casualty Phenomenon.pptxInstructions for Residents.docxStudent Small-Group Prompts.docxCoping with Complications.pptxStudent Role-Play Instructions.docxWorkshop Facilitator Guide and Schedule.docx [file mep_2374-8265.11473-s001.zip › D. Student Small-Group Prompts.docx]

***Students should be broken up into groups of 3 or 4. This sheet can be handed out to students to help prompt discussion.***

(Questions to prompt discussion in first small group)

- What is the most intense patient harm event you have been involved in?
- If you weren’t involved in an event, have you seen one while on service?
- How did it seem to affect the team members?
- What coping mechanisms have you used to deal with stressful clinical events?
- How have you seen interns and residents coping with patient harm events? Do certain coping mechanisms appear to be more successful as compared to others?
- Do you think debriefing or M&M sessions are helpful? Why or why not?
