# Supplementary material for: From Healer to Harmer: Preparing Senior Medical Students for Patient Harm Events in a Transition-to-Residency Course
Source: MedEdPORTAL. 2024 Dec 26;20:11473. doi: 10.15766/mep_2374-8265.11473 (PMC11669734; doi:10.15766/mep_2374-8265.11473)
Supplement: Supplementary file 1 — Pre- and Postsurvey.docxSecond Casualty Phenomenon.pptxInstructions for Residents.docxStudent Small-Group Prompts.docxCoping with Complications.pptxStudent Role-Play Instructions.docxWorkshop Facilitator Guide and Schedule.docx [file mep_2374-8265.11473-s001.zip › F. Student Role-Play Instructions.docx]

**Facilitator Instructions:**

*Facilitators should pair up students into groups of two. Each pair should be assigned a role as Student A and Student B. For the first 7 minutes Medical Student A is the senior resident and Medical Student B is the junior resident. Halfway through, the medical students should switch roles for the second scenario. Medical Student A should now be the junior resident and Medical Student B should be the senior resident for the second 7 minutes.*

**Student Hand-out: Role Play Student A**

Role Play Scenario #1

You are the junior resident. You have been following a patient, Mrs. Hernandez, who was just transferred to the ICU because she required intubation. You think back to the events leading up to the transfer and remember the RN calling you twice about Mrs. Hernandez “looking worse”. When you received those calls, you had been busy with another decompensating patient – you didn’t prioritize evaluating her until a rapid response was called. You wonder if you had evaluated her sooner, whether intubation could have been avoided.

*Your task is to alert your senior resident of Mrs. Hernandez’ escalation of care and reflect on your potential contribution to her worsening respiratory status.*

You as the junior resident start the conversation: “Hey chief, I just wanted to let you know the patient I have been taking care of today is going to the ICU.”

- Sample follow-up prompts for you to use:
  - “I really feel like I should have done more. This is all my fault.”
  - “I should have checked on her when they first called, I was just so busy…”
  - “I’m worried that I won’t ever graduate residency if things like this keep happening while I’m on service.”

*Switch Roles after 7 minutes.*

Role Play Scenario #2

You are now the senior resident. Earlier today, you supervised the junior resident in placing a central line on an intubated patient in the ICU. The central line was placed with ultra-sound, without difficulty. The post-procedure X-ray shows a pneumothorax, which surprised you given the proper technique and easy anatomy. After clinically addressing the pneumothorax, you and the junior resident are now in the workroom, completing documentation for the day.

*Wait for the junior to start the conversation.*

*Your task is to de-brief the junior resident from this complication in a judgment-free, supportive environment that allows the junior resident to cope from this experience.*

***Guiding Principle: Utilize the Royal College of Surgeons “First Responder” framework to de-brief the event.***

**Student Hand-out: Role Play Student B**

*Pair up in groups of 2 learners.*

Role Play Scenario #1

You are the senior resident supervising a junior resident. The junior resident had been managing a patient, Mrs. Hernandez, who was just transferred to the ICU because she required intubation. You know little about the patient’s case; the junior hadn’t alerted you to any concerns or needing any help – until now.

*Wait for the junior to start the conversation.*

*Your task is to de-brief the junior resident from this complication in a judgment-free, supportive environment that allows the junior resident to learn from the experience.*

***Guiding Principle: Utilize the Royal College of Surgeons “First Responder” framework to de-brief the event.***

*Switch Roles after 7 minutes.*

Role Play Scenario #2:

You are now the junior resident. Earlier today, you placed a central line on an intubated patient in the ICU. Your senior resident supervised the entire procedure; both of you were pleased with how smoothly the procedure went. Now, the post-procedure X-ray shows there is a pneumothorax. After clinically addressing the pneumothorax, you and the senior resident are in the workroom, completing documentation for the day.

You, as the junior resident: “I feel terrible that I caused a pneumothorax. I’m such a horrible resident.”

*Your task is to alert your senior resident that you feel completely responsible for this complication. As you work through the example, your goal over time is to realize that procedural complications can occur even with the proper technique.*

- - Sample follow up prompts for you, the junior resident, to use:
    - “I worry the attendings are going to think I’m incompetent.”
    - “Is it normal for something like this to happen?”
    - “Should we talk to the patient’s family?”
