# Supplementary material for: From Healer to Harmer: Preparing Senior Medical Students for Patient Harm Events in a Transition-to-Residency Course
Source: MedEdPORTAL. 2024 Dec 26;20:11473. doi: 10.15766/mep_2374-8265.11473 (PMC11669734; doi:10.15766/mep_2374-8265.11473)
Supplement: Supplementary file 1 — Pre- and Postsurvey.docxSecond Casualty Phenomenon.pptxInstructions for Residents.docxStudent Small-Group Prompts.docxCoping with Complications.pptxStudent Role-Play Instructions.docxWorkshop Facilitator Guide and Schedule.docx [file mep_2374-8265.11473-s001.zip › G. Workshop Facilitator Guide and Schedule.docx]

Facilitator Guide and Schedule

5 minutes | Introduction

- - Ignite curiosity for the topic of patient complications and set the tone of vulnerability

15 minutes | Didactic session on second casualty phenomenon.

- - Reference PowerPoint (Appendix B)

20 minutes | Resident panel

*Faculty facilitator & 2-3 resident physicians share patient harm events and coping strategies*

- - If you are comfortable being vulnerable, recommend sharing your own experience with a patient harm event as an icebreaker. You should then invite the residents to share their experiences with patient harm events. (Appendix C)
  - Ask each resident to narrate the story of the patient harm event and their perceived role in the event. Guide each resident to address three key areas after their story:
    - Emotional impact: "How did this event affect you emotionally?"
    - Coping strategies: "What methods did you use to handle these emotions?"
    - Lessons learned: "What insights did you gain for managing future patient harm events?"
  - Additional questions for resident panel if time allows:
    - Did you find debriefing or M&M helpful for coping with patient harm events?

15 minutes | Small group discussion: students discuss complications and coping mechanisms

- - Use (Appendix D) for instructions.
  - If students do not want to discuss any patient harm events they have directly been involved in they can use patient harm events they have witnessed in the discussion.

10 minutes | Large group de-brief: general exposure to patient harm events

- - Invite students to share experiences with patient harm events they've been involved in or witnessed. Emphasize this is voluntary.
  - For students who share, ask “How did you cope with this event emotionally? How did you witness others on your team cope with this event emotionally? What helped or didn’t help?”
  - Validate students’ feelings about these events. Say: “These experiences are common and are challenging. Has anyone else faced something similar?”
  - If no students respond, ask how they have seen residents deal with patient harm events
  - Move on to second didactic session once discussion has ended

15 minutes | Didactic session: Coping strategies and how to help others through complications

- - Reference PowerPoint on Coping Strategies (Appendix E)
  - Leave slides on First Responder Framework on screen for reference during role play

15 minutes | Scenario role play

- - Ask students to get in pairs. Facilitator to hand out (Appendix F) to pairs of students.
  - The facilitator can move between groups of students to answer questions during this time.
- 10 minutes | De-brief after role play exercise
  - Ask students to share their experience roleplaying helping a colleague cope with a patient harm event
  - Ask students to discuss how the two scenarios were different and if that changed how they counseled the junior resident. Some patient harm events are “expected complications” (pneumothorax after a central line) whereas other complications may be more associated with a provider mistake (difficulty triaging patient severity).
  - Have students anticipate potential challenges in debriefing a colleague after a patient harm event
  - Explore how openly addressing these challenges can benefit team members and overall team dynamics
- 5 minutes | Closing and questions
