# Supplementary material for: From Healer to Harmer: Preparing Senior Medical Students for Patient Harm Events in a Transition-to-Residency Course
Source: MedEdPORTAL. 2024 Dec 26;20:11473. doi: 10.15766/mep_2374-8265.11473 (PMC11669734; doi:10.15766/mep_2374-8265.11473)
Supplement: Supplementary file 1 — Pre- and Postsurvey.docxSecond Casualty Phenomenon.pptxInstructions for Residents.docxStudent Small-Group Prompts.docxCoping with Complications.pptxStudent Role-Play Instructions.docxWorkshop Facilitator Guide and Schedule.docx [file mep_2374-8265.11473-s001.zip › _Educational Summary Report_11473.pdf]

MedEdPORTAL® | The AAMC Journal of Teaching and Learning Resources

Original Publication

Open Access

From Healer to Harmer: Preparing Senior Medical Students for Patient Harm Events in a Transition-to-Residency Course

Campbell Grant, MD*, Sabina Warns, Lillian Sims, PhD, Kristen E. Fletcher, MD

*Corresponding author: campbell.grant@uky.edu

Abstract

Introduction: A physician's first patient harm event oftentimes occurs during the intern year. Residents encounter and are responsible for medical errors, yet little training is offered in how to properly cope with these events. Earlier and more in-depth education about how to process patient harm events is needed. **Methods:** We developed a 110-minute workshop focused on coping strategies for patient harm events and delivered it to a cohort of fourth-year medical students during a transition-to-residency course just before graduation. The workshop emphasized interns' increasing exposure to medical errors, how to personally process them, and how to debrief near-peers in processing them. **Results:** A total of 190 students participated in the workshop. Our survey response rate was 88%. Students' confidence in defining second casualty after the workshop grew from eight responding *very* or *extremely confident* (7%) to 95 responses (87%). Comfort utilizing positive coping mechanisms improved from 14 *very* or *extremely confident* responses (12%) to 73 responses (67%). Confidence utilizing first responder structure grew from three *very* or *extremely confident* responses (3%) to 61 responses (56%). Comfort helping colleagues cope with patient harm events grew from 16 *very* or *extremely confident* responses (14%) to 78 responses (72%). **Discussion:** This workshop fills an important gap in UME by preparing senior-level students to resolve emotional conflict related to patient harm events. Our findings illustrate that a short-term intervention on this topic can impact students' confidence. We believe discussion around how patient harm events emotionally impact trainees should be expanded.

Keywords
Transition to Residency, Complications, Coping Skills, Patient Harm

Educational Objectives

By the end of this activity, learners will be able to:

1. Define second casualty phenomenon.
2. Categorize coping strategies as adaptive or maladaptive.
3. Apply a healthy coping mechanism framework to patient complications.
4. Utilize debriefing techniques to support a near-peer colleague following a patient complication.

Introduction

During the last 2 decades, patient safety and trainee well-being curricula have been increasingly introduced into medical education alongside widespread discussion of physician burnout and associated risks of medical error.[1-3] At the intersection of patient safety and trainee well-being lies the emotional recovery of the physician involved with causing patient harm, perhaps for the first time. Medical students, while regularly exposed to concepts of patient safety and error prevention, have described intense emotional reactions to the idea of their errors causing patient harm, yet no published curriculum exists to help prepare them for patient harm events.[4] Resident physicians also describe intense emotional reactions to harm events.[5] The ACGME has developed the Clinical Learning Environment Review Pathways to Excellence, which exposes residents and fellows to patient safety and quality improvement principles, but the primary focus is on error identification and prevention, not on emotional recovery.[6] Meanwhile, research on second casualty phenomenon (originally referred to as second victim phenomenon) has focused on the emotional risks to health care workers involved in patient harm events after they occur, with minimal proactive training.[7]

Citation:
Grant C, Warns S, Sims L, Fletcher KE. From healer to harmer: preparing senior medical students for patient harm events in a transition-to-residency course. *MedEdPORTAL*. 2024;20:11473.
https://doi.org/10.15766/mep_2374-8265.11473

Despite increased awareness, fully trained physicians continue to struggle with the emotional burden of complications.[8] There are significant individual and systemic benefits associated with better training for managing patient harm events. Healthy processing of

Copyright © 2024 Grant et al. This is an open-access publication distributed under the terms of the Creative Commons Attribution license.

patient harm can facilitate emotional recovery, potentially leading to less burnout of both physicians in training and attending physicians.

UME courses have been designed to improve resilience of medical students as they transition from a teaching environment to a work environment. However, these courses do not directly address how trainees can cope with the emotional burden associated with patient harm.[9] Teaching medical students how to anticipate and emotionally recover from harm events is necessary because for the first time in their training, resident physicians are poised to have responsibility where medical errors do occur. Resident physicians' serious medical error rates have been well studied, especially surrounding 2003 duty hour regulatory changes, and resident physicians commonly self-perceive causing medical errors.[10] One study found that 23% of interns self-reported committing a serious medical error, and, in fact, the majority of medical errors occur during the intern year.[11,12]

When resident physicians perceive that they have caused patient harm events, the impact on the individual physician's well-being is substantial and includes greater depersonalization, emotional exhaustion, and decreased quality of life.[13,14] In turn, resident physicians suffering from depersonalization and emotional exhaustion are at greater risk for future self-perceived major patient harm events,[14] thus making resident distress and patient harm events a reciprocal cycle.[13] In research on patient harm curricula, residents have expressed a desire to have a forum in which they can learn to manage the emotions involved in errors and their own responses.[15] Thus, curricula focusing on the emotional distress of one patient harm event may help prevent another patient harm event from occurring by breaking this cycle.

Current literature on the curriculum for medical students and resident physicians works to mitigate the risk of patient harm events by emphasizing systematic quality improvement and organizational culture.[15,16] Meanwhile, the emotional recovery process for individual resident physicians involved with a patient harm event, specifically those at the transition-to-residency (TTR) stage, has yet to be adequately addressed either in research literature or in curricula. Although educational innovations have addressed how residents could or should respond to patient harm events,[17] and wider attention to the emotional burden of errors remains warranted,[18] we posit that emotional recovery needs to be emphasized systematically during UME and, in particular, during TTR curricula to prepare trainees to use appropriate communication and coping skills before they enter the high-exposure context of residency. To address this need, we developed a workshop focused on trainee emotional recovery after patient harm and integrated it within a senior-level course. To our knowledge, this is one of the first courses to directly and formally address how to emotionally manage complications caused during training, and we expect that greater integration of this content across institutions could have a powerful protective effect for graduating physicians.

Methods

Course Design

We developed content for the workshop drawing on our experiences during the supervision of medical and surgical interns, noting their emotional distress over their first patient harm events. Informal focus groups with resident physicians within our clinical departments highlighted this educational need. We met monthly on average for 9 months preceding the course to discuss the literature underpinning the educational gap and to develop the best-fit pedagogy.

We ultimately used a 110-minute communications workshop as the core instructional methodology, centered on small-group reflection, role-play, and a resident panel. This workshop was rooted in the educational framework of transformative learning, where students' physician identity formation is challenged from healer to potential harmer.[19] The workshop had several components: a presession survey (Appendix A), a didactic introduction to second casualty phenomenon (Appendix B), a resident panel (Appendix C) and a subsequent small-group discussion (Appendix D), a didactic introduction to coping mechanisms (Appendix E), a role-play session (Appendix F), and a postsession survey (Appendix A). A facilitator guide (Appendix G) was included to help physicians administer the course at satellite campuses. The survey was designed by the course directors to assess students' initial familiarity with the topic and the effectiveness of the workshop.

The objective of the resident panel was to foster acceptance among students that patient harm events occur with regularity in clinical practice, to emphasize such events are an inevitable aspect of their future careers as physicians, and to underscore the benefits of engaging in structured debriefing with colleagues, such as the Royal College of Surgeons (RCS) framework, rather than processing these incidents through solitary reflection. Alternatively, the workshop could be conducted using faculty panelists describing their experiences; however, we felt that resident physicians would be more relatable to undergraduate learners. The role-play session leaned on a framework for healthily debriefing complications, which was developed by RCS.[20] This framework was originally envisioned as part of a quality improvement project to minimize the impact of an

Copyright © 2024 Grant et al. This is an open-access publication distributed under the terms of the Creative Commons Attribution license.

adverse event on surgeons while supporting patient safety. Beyond its surgical origins, the content creates a general framework for any medical professional to safely discuss a patient harm event, which was why we built upon it for our role-play scenarios. Our inclusion of peer debriefing skills was based on the widespread discussion that occurs between the resident physician who made a mistake and another resident physician. In fact, resident physicians debrief more commonly with another resident physician (83%) compared to any other group, including supervising faculty, with whom they discuss the case only 54% of the time.[13] Despite the commonality of peer debriefing for patient harm events, there is limited training performed across the UME to GME spectrum for resident physician recipients of such delicate matters.[21]

Setting

We designed a curriculum for fourth-year medical students within our existing TTR course. This 4-week senior capstone course teaches clinical, communication, and physician identity formation skills needed for the successful transition to internship through workshops and simulation. Our TTR course occurs each April and is a requirement for graduation. The Overcoming Complications workshop we describe here was first delivered in April 2023; every fourth-year medical student at our institution participated in the session. Our College of Medicine includes a large main campus, two regional campuses, and one regional site, with a 2023 graduating class of 192.

Faculty Preparation

The logistics of sharing content across multiple campuses with differing faculty and resources are complex. We recruited physicians from regional campus sites to facilitate learning at their local sites. All faculty recruited were volunteers. We held a training course 2 weeks prior to prepare faculty to facilitate sessions across sites. At the main campus, the course educator who developed the content led each version of the workshop. The training course entailed a 1-hour videoconference where we guided each faculty through the presentations that they would give as well as the role-play scenarios (Appendices B-F) and their jobs as facilitators. We also recruited residents across several specialties who were identified by their program directors because of their willingness to be vulnerable with other trainees about their experiences.

Delivery Process

We delivered the workshop in four separate iterations for the main campus with 30-35 students in each session to maintain a small-group learning environment. Additionally, the workshop was delivered in a single session to each of our two regional campuses and one regional clinical site. On the main campus, each group was organized by specialty, such that all students going into surgical specialties were grouped together, all students going into medical specialties were grouped together, etcetera. Each session lasted approximately 110 minutes and included the following:

- 5-minute introduction
- 15-minute didactic session on second casualty phenomenon (Appendix B)
- 20-minute resident panel: one faculty facilitator with two to three resident physicians discussing patient harm events they were involved in as well as coping skills they used (Appendix C)
- 15-minute small-group discussion amongst students on complications and coping mechanisms (Appendix D)
- 10-minute large-group debrief: students asked to share their experiences with patient harm events
- 15-minute didactic session on coping strategies and how to help others through complications (Appendix E)
- 15 minutes of scenario role-play between pairs of two students (Appendix F)
- 10-minute debrief after role-play exercise
- 5 minutes of closing and questions

Resources

The following resources were required to implement the workshop: (1) educational materials, (2) one faculty facilitator per site, (3) two to three resident physicians (or the most junior physicians available in the community), and (4) a classroom location that could accommodate evenly spaced small groups.

We assessed the effectiveness of our activity with a pre- and postsurvey. The University of Kentucky Institutional Review Board (IRB) approved this project (protocol #81351).

Results

A total of 190 students across four campuses completed the workshop, which was built into the required fourth-year TTR course. Of the participants, 128 completed the pretest and 113 completed the posttest. Participant data collection was limited to the main campus cohort for the initial pilot year described here. Feedback from faculty facilitators at regional campuses was positive and plans are in place to continue the workshop at each campus during TTR.

Our total survey response rate was 88%. Students' confidence in defining second casualty grew pre- to postsurvey from eight responding *very* or *extremely confident* (7% of presurvey takers) to 95 responses (87%; Figure 1). Comfort utilizing positive

Copyright © 2024 Grant et al. This is an open-access publication distributed under the terms of the Creative Commons Attribution license.

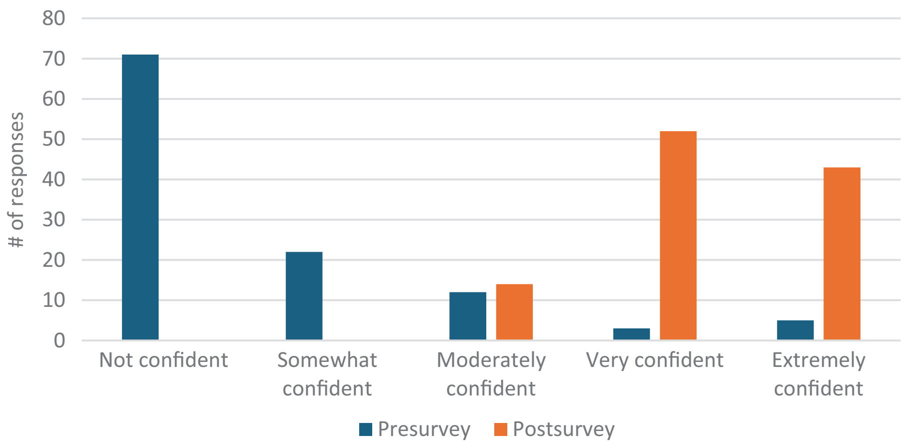

Figure 1. Students' confidence in defining the second casualty phenomenon before and after the workshop.

coping mechanisms improved from 14 *very* or *extremely confident* responses (12%) to 73 responses (67%; Figure 2). Confidence utilizing first responder structure grew from three *very* or *extremely confident* responses (3%) to 61 responses (56%; Figure 3). Finally, comfort helping colleagues cope with patient harm events grew from 16 *very* or *extremely confident* responses (14%) to 78 responses (72%; Figure 4). Data analysis using a paired *t* test shows a significant ($p = .001$) increase in students responding either *very* or *extremely confident* for all four categories.

Overall, the students appear to be more confident in their ability to define and cope with patient harm events. These results fall under Kirkpatrick level one of reaction,[22] as we are unable to track currently if students are using these skills after graduation. Thus, we view these data as timely and useful for considering additions or modifications to UME curricula, but also potentially increasingly valuable with long-term follow-up as participants enter residency.

Discussion

Complications and patient harm events are omnipresent in clinical practice. This workshop fills an important gap[4] in UME by directly preparing senior-level students to encounter patient harm events on a personal level. These findings illustrate how even a short-term intervention on this topic can have a measurable impact on students' confidence about navigating patient harm events prior to entering residency. We expect that the choice to place this content in a fourth-year TTR course maximizes its usefulness to trainees who are soon to enter residency. Intern year is often where trainees experience the largest jump in patient care responsibility. The sudden increase of patient ownership and accountability can contribute to feelings of anxiety

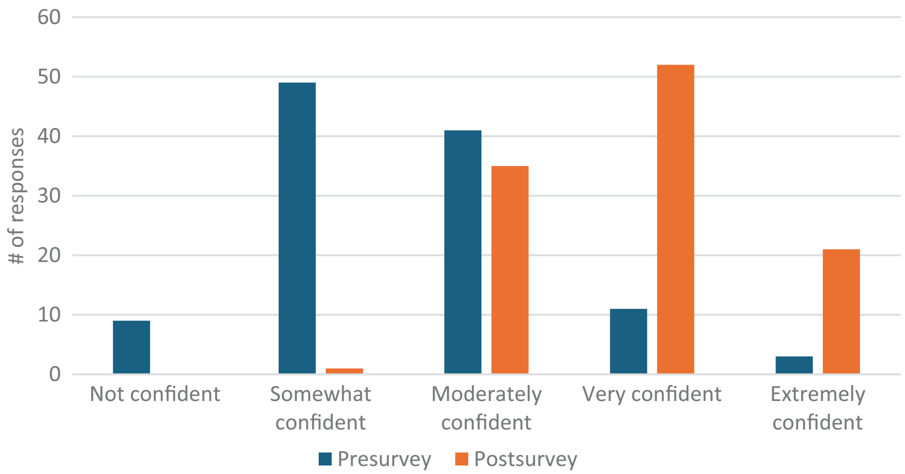

Figure 2. Students' confidence in utilizing positive coping mechanisms before and after the workshop.

Copyright © 2024 Grant et al. This is an open-access publication distributed under the terms of the Creative Commons Attribution license.

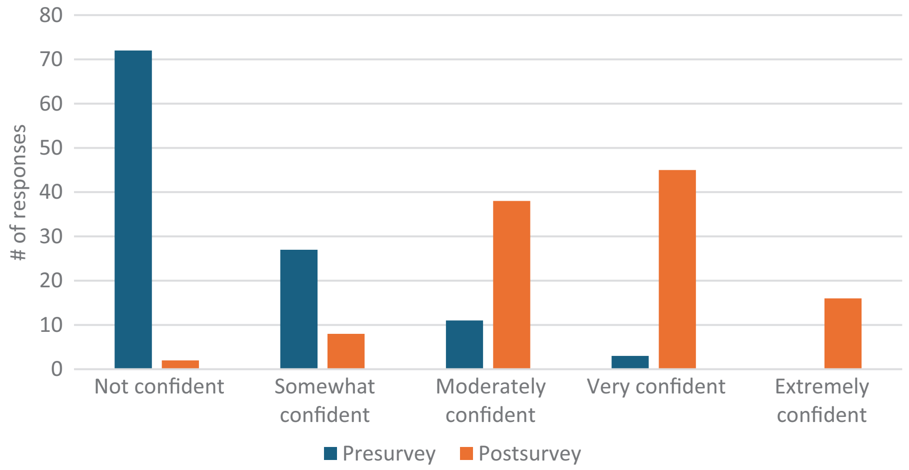

Figure 3. Students' confidence in using the first responder structure for discussing patient harm events before and after the workshop.

and guilt when patient harm events occur. In addition to the educational impact of the content itself, we theorize that the act of openly discussing different aspects of this issue (e.g., coping strategies; supporting colleagues) may destigmatize seeking help for emotionally complex case management and have benefits for both students and residents involved. Our results indicate that despite training about medical error more generally, students had very limited exposure to the specific topic of emotional preparation for and recovery from patient harm events prior to our course. Discussion around this topic was previously only in informal settings and not ensured for all students, and more broadly, focused on residents and not students.[23] By formalizing this content but maintaining the small-group, open-discussion format suiting the topic, we ensured better preparation of all our trainees.

A strength of this workshop is its generalizability; the ubiquity of patient harm events makes the topic relatable to most clinical faculty, allowing a large pool of potential clinical faculty members to teach the workshop. Classroom resource requirements are standard, and few time-intensive preparations are necessary. While we taught this workshop to senior medical students during TTR, this workshop could be easily adapted to intern orientation, resident teaching sessions, or faculty development settings.

Another strength was the cathartic emotional impact of this workshop, not just for students but for the resident physician panelists. General feedback for each workshop within the larger TTR course was collected from all students participating to assess the effectiveness of the course. Course feedback for our course was collected only from the main campus because

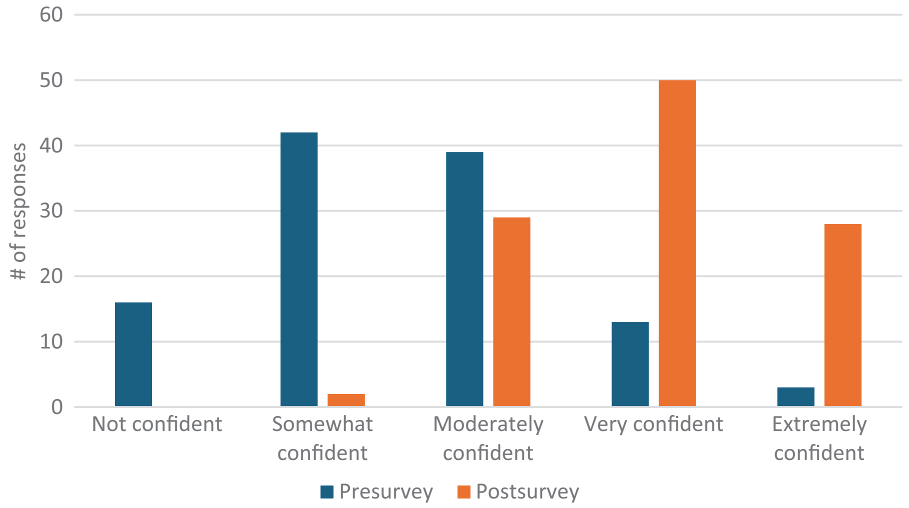

Figure 4. Students' confidence in helping colleagues cope with patient harm events before and after the workshop.

Copyright © 2024 Grant et al. This is an open-access publication distributed under the terms of the Creative Commons Attribution license.

of how faculty are reviewed within the course. Feedback was overall very positive. For the question “Was this an effective course?,” 75 students responded with a mean score of 3.8 out of 4. The following representative comment was written in the course evaluation: “I really appreciated the open discussion, which encouraged a lot of the students to speak up about their own experiences.”

Several of the resident panelists became emotional while sharing narratives surrounding patient harm events in the small-group setting. Resident panelists recalled a heightened feeling of imposter phenomenon for weeks following a specifically difficult patient harm event. Formal qualitative data were not collected from the resident physician panelists; however, in debriefing their panelist experience, many resident panelists shared they were surprised their colleagues felt similar shame in having their care lead to a patient harm event. The reaction by the residents underscores the need to start these conversations earlier and continue them more robustly throughout clinical training. Residents mentioned at the end of the session they had not previously had a forum to emotionally process the cases they were called upon to discuss. Overall, this finding aligns with other research supporting the value of involving residents in UME as near-peers who can both support new trainees and find personal growth in doing so.[24]

Throughout this workshop, we learned that many students had not previously felt any responsibility for patient harm events that occurred while they were rotating on service. Furthermore, students often had a hard time recalling patient harm events even occurring while they were on service. This finding is substantiated in other literature, where only 36% of medical students reported witnessing a patient harm event during their UME.[25] In our view, this highlights the major shift of increased accountability and awareness of patient harm events that fourth-year students must be prepared to undergo as they enter residency. We hypothesize that residents and attending physicians are shielding students from the negative impacts that patient harm events cause to minimize negative feelings. This shielding strategy may accidentally be teaching students that health care teams do not openly discuss the emotional impact of patient harm events, especially when residents or attending physicians involved do not include students in reflection. This strategy may have short-term protective benefits for students but ensures the need for focused preparation for residency-stage impacts of patient harm events like this workshop.

Challenges we faced include teaching across multiple, diverse campuses (which complicates access and quality control) and timing, with the workshop limited in longitudinal scope due to its placement in a single fourth-year course. In terms of evaluation, our primary initial limitation is a lack of insight into the extent to which trainees modified their approaches or reactions to patient harm events once they entered residency. Our pre/postsurvey design allowed for short-term awareness of the innovation’s potential impact, which allows us to build and revise the workshop year-to-year. However, we were unable to collect survey data from our regional campuses, which limits our ability to measure the effectiveness of the course across sites. Long term, our IRB protocol allows for follow-up interviews with workshop participants once they are firmly established as residents in a range of GME programs. We anticipate that longitudinal data will yield additional insight into remaining needs relating to this topic, whether at the UME or GME level or specific to certain specialties or contexts.

Emerging medical education literature recognizes the need to prepare medical students for emotional recovery from involvement in patient harm events.[4] We anticipate that incorporating this workshop into fourth-year TTR curricula can enable UME programs to lay a foundation for greater awareness, discussion, and support for trainees who may soon be encountering patient complications for which they feel primary responsibility. The session, in addition to the evaluation procedure, can provide institutionally specific feedback about additional needs relating to this topic and can help leadership improve support for trainees who may struggle adjusting to the emotional burden that comes with increased clinical responsibility and risk.

Appendices

A. Pre- and Postsurvey.docx
B. Second Casualty Phenomenon.pptx
C. Instructions for Residents.docx
D. Student Small-Group Prompts.docx
E. Coping with Complications.pptx
F. Student Role-Play Instructions.docx
G. Workshop Facilitator Guide and Schedule.docx

All appendices are peer reviewed as integral parts of the Original Publication.

Campbell Grant, MD: Assistant Professor, Department of Urology, University of Kentucky College of Medicine; ORCID: https://orcid.org/0000-0002-3425-0556

Copyright © 2024 Grant et al. This is an open-access publication distributed under the terms of the Creative Commons Attribution license.

Sabina Warns: Third-Year Medical Student, University of Kentucky College of Medicine

Lillian Sims, PhD: Assistant Professor, Department of Behavioral Science, University of Kentucky College of Medicine

Kristen E. Fletcher, MD: Associate Professor, Department of Academic Medical Education and Medicine, University of Kentucky College of Medicine and Lexington Veterans Affairs Health Care

Disclosures
None to report.

Funding/Support
None to report.

Prior Presentations
Warms S, Sims L, Fletcher K, Grant C. From MS4 to intern and healer to harmer. Presented virtually at: Transition to Residency Symposium; October 3, 2024.

Ethical Approval
The University of Kentucky Institutional Review Board approved this project.

References

1. Shanafelt TD, Balch CM, Bechamps G, et al. Burnout and medical errors among American surgeons. *Ann Surg*. 2010;251(6): 995-1000. https://doi.org/10.1097/SLA.0b013e3181bfdab3
2. Tawfik DS, Profit J, Morgenthaler TI, et al. Physician burnout, well-being, and work unit safety grades in relationship to reported medical errors. *Mayo Clin Proc*. 2018;93(11): 1571-1580. https://doi.org/10.1016/j.mayocp.2018.05.014
3. West CP, Dyrbye LN, Shanafelt TD. Physician burnout: contributors, consequences and solutions. *J Intern Med*. 2018;283(6):516-529. https://doi.org/10.1111/joim.12752
4. Krogh TB, Mielke-Christensen A, Madsen MD, Østergaard D, Dieckmann P. Medical students' experiences, perceptions, and management of second victim: an interview study. *BMC Med Educ*. 2023;23:786. https://doi.org/10.1186/s12909-023-04763-7
5. Fischer MA, Mazor KM, Baril J, Alper E, DeMarco D, Pugnaire M. Learning from mistakes: factors that influence how students and residents learn from medical errors. *J Gen Intern Med*. 2006; 21(5):419-423. https://doi.org/10.1111/j.1525-1497.2006.00420.x
6. *CLER Pathways to Excellence: Expectations for an Optimal Clinical Learning Environment to Achieve Safe and High Quality Patient Care*. Accreditation Council for Graduate Medical Education; 2014. Accessed November 12, 2024. https://www.acgme.org/globalassets/PDFs/CLER/CLER_Brochure.pdf
7. Scott SD. The second victim phenomenon: a harsh reality of health care professions. Agency for Healthcare Research and Quality. May 1, 2011. Accessed November 12, 2024. https://psnet.ahrq.gov/perspective/second-victim-phenomenon-harsh-reality-health-care-professions
8. Vitous CA, Byrnes ME, De Roo A, Jafri SM, Suwanabol PA. Exploring emotional responses after postoperative complications: a qualitative study of practicing surgeons. *Ann Surg*. 2022; 275(1):e124-e131. https://doi.org/10.1097/SLA.0000000000004041
9. Bird A, Tomescu O, Oyola S, Houpy J, Anderson I, Pincavage A. A curriculum to teach resilience skills to medical students during clinical training. *MedEdPORTAL*. 2020;16:10975. https://doi.org/10.15766/mep_2374-8265.10975
10. Landrigan CP, Rahman SA, Sullivan JP, et al; ROSTERS Study Group. Effect on patient safety of a resident physician schedule without 24-hour shifts. *N Engl J Med*. 2020;382(26):2514-2523. https://doi.org/10.1056/NEJMoa1900669
11. Sen S, Kranzler HR, Didwania AK, et al. Effects of the 2011 duty hour reforms on interns and their patients: a prospective longitudinal cohort study. *JAMA Intern Med*. 2013;173(8): 657-662. https://doi.org/10.1001/jamainternmed.2013.351
12. Fatima S, Soria S, Esteban-Cruciani N. Medical errors during training: how do residents cope?: a descriptive study. *BMC Med Educ*. 2021;21:408. https://doi.org/10.1186/s12909-021-02850-1
13. West CP, Huschka MM, Novotny PJ, et al. Association of perceived medical errors with resident distress and empathy: a prospective longitudinal study. *JAMA*. 2006;296(9):1071-1078. https://doi.org/10.1001/jama.296.9.1071
14. West CP, Tan AD, Habermann TM, Sloan JA, Shanafelt TD. Association of resident fatigue and distress with perceived medical errors. *JAMA*. 2009;302(12):1294-1300. https://doi.org/10.1001/jama.2009.1389
15. Szymusiak J, Fox MD, Polak C, et al. An inpatient patient safety curriculum for pediatric residents. *MedEdPORTAL*. 2018;14: 10705. https://doi.org/10.15766/mep_2374-8265.10705
16. Garcia C, Goolsarran N. Learning from errors: curriculum guide for the morbidity and mortality conference with a focus on patient safety concepts. *MedEdPORTAL*. 2016;12:10462. https://doi.org/10.15766/mep_2374-8265.10462
17. Bari A, Khan RA, Rathore AW. Medical errors; causes, consequences, emotional response and resulting behavioral change. *Pak J Med Sci*. 2016;32(3):523-528. https://doi.org/10.12669/pjms.323.9701
18. White AA, Gallagher TH. After the apology: coping and recovery after errors. *Virtual Mentor*. 2011;13(9):593-600. https://doi.org/10.1001/virtualmentor.2011.13.9.ccas1-1109
19. Vipler B, Knehans A, Rausa D, Haidet P, McCall-Hosenfeld J. Transformative learning in graduate medical education: a scoping

Copyright © 2024 Grant et al. This is an open-access publication distributed under the terms of the Creative Commons Attribution license.

review. *J Grad Med Educ*. 2021;13(6):801-814. https://doi.org/10.4300/JGME-D-21-00065.1

20. Supporting surgeons after adverse events. Royal College of Surgeons of England. Accessed November 12, 2024. https://www.rcseng.ac.uk/standards-and-research/standards-and-guidance/good-practice-guides/supporting-surgeons-after-adverse-events/guide/
21. Seitz KP, Baclig NV, Stiller R, Chen A. Implementation of a near-peer support program to improve trainee well-being after patient safety events. *ATS Sch*. 2023;4(4):423-430. https://doi.org/10.34197/ats-scholar.2023-0011PS
22. Kirkpatrick JD, Kirkpatrick WK. *Kirkpatrick's Four Levels of Training Evaluation*. ATD Press; 2016.
23. Engel KG, Rosenthal M, Sutcliffe KM. Residents' responses to medical error: coping, learning, and change. *Acad Med*. 2006; 81(1):86-93. https://doi.org/10.1097/00001888-200601000-00021
24. Raty SR, Teal CR, Nelson EA, Gill AC. Near-peers improve patient safety training in the preclinical curriculum. *Med Educ Online*. 2017;22(1):1289315. https://doi.org/10.1080/10872981.2017.1289315
25. Kiesewetter J, Kager M, Lux R, Zwissler B, Fischer MR, Dietz I. German undergraduate medical students' attitudes and needs regarding medical errors and patient safety—a national survey in Germany. *Med Teach*. 2014;36(6):505-510. https://doi.org/10.3109/0142159X.2014.891008

Received: January 4, 2024
Accepted: September 12, 2024
Published: December 26, 2024

Copyright © 2024 Grant et al. This is an open-access publication distributed under the terms of the Creative Commons Attribution license.
